# Prevalence of fall risk and fear of falling among older adults in the Middle East and North Africa: a systematic review and meta-analysis

**DOI:** 10.7189/jogh.16.04397

**Published:** 2026-10-30

**Authors:** Karima Chaabna, Anupama Jithesh, Salina Khawaja, Jasmine Aboughanem, Ravinder Mamtani, Sohaila Cheema

**Affiliations:** Global Institute for Population Health, Weill Cornell Medicine-Qatar, Doha, Qatar

**Keywords:** fall risk, fear of falling, older adults, Middle East, North Africa

## Abstract

**Background:**

Globally, falls are a major cause of morbidity and mortality in older adults (individuals aged ≥60 years). In the Middle East and North Africa (MENA) region, home to over 57 million older adults, data on key fall-related risk factors are limited, constraining targeted prevention efforts. We aimed to estimate the prevalence of fall risk and fear of falling among older adults in the 20 MENA countries.

**Methods:**

We searched PubMed, Web of Science, Google Scholar, and Google (up to November 2025). Two independent reviewers screened studies, extracted data, and assessed quality. We estimated pooled prevalence using random-effects meta-analysis and conducted meta-regression to explore heterogeneity.

**Results:**

We included 109 studies with 213,877 older adults. The mean age ranged from 63.1 (standard deviation (SD) = 2.5) to 77.2 (SD = 4.6) years across studies. The pooled prevalence of older adults at higher fall risk was 54.2% (95% confidence interval (CI) = 35.1, 72.8) based on the Missouri Alliance for Home Care Fall Risk Assessment and 33.4% (95% CI = 18.4, 53.8) based on the Morse Fall Scale. The pooled prevalence of older adults classified at higher fall risk was 41.7% (95% CI = 27.6, 57.2) based on balance performance using the Berg Balance Scale and 58.0% (95% CI = 39.4, 74.5) based on functional mobility using the Timed Up and Go Test. Studies reporting fear of falling mainly used validated tools assessing concern about falling and fall-related self-efficacy. The pooled prevalence of moderate to high concern about falling, measured by the Falls Efficacy Scale–International, was 82.7% (95% CI = 60.4, 93.8). The pooled prevalence of reduced fall-related self-efficacy, assessed by the Modified Falls Efficacy Scale, was 67.1% (95% CI = 44.9, 83.6). We observed substantial heterogeneity across studies, with no significant differences by population type, sex, or country-income level in meta-regression analyses. We detected no evidence of publication bias for fear of falling, while minor asymmetry was observed for fall risk; however, data were unavailable for ten MENA countries.

**Conclusions:**

This review shows a substantial prevalence of fall risk and fear of falling among older adults in the MENA countries represented in the available evidence, underscoring the need for region-specific fall prevention interventions. These findings support integrating routine fall-risk screening and standardised assessment tools into primary and geriatric care to address both physical and psychological risk factors and to inform targeted interventions across the region.

**Registration:**

Open Science Framework: https://osf.io/3cu4q/overview

Falls are a leading cause of morbidity and mortality among older adults [1], with an estimated 172 million falls occurring annually worldwide in this population [2]. Falls frequently result in substantial physical injury, psychological trauma, and loss of independence, often leading to disability and hospitalisation [3,4]. The cumulative impact of falls extends beyond affected individuals, placing a significant burden on caregivers and healthcare systems [2].

Effective fall risk assessment requires validated, setting-appropriate tools, as risk profiles vary across hospitals, long-term care, and community settings [5]. The methodological quality of the evidence depends on selecting tools suited to the target population, ensuring linguistic and cultural relevance, and reporting transparently. Fall risk is multifactorial, with mobility assessment representing a core domain that includes balance, gait, functional mobility, and the need for walking aids [4]. Fear of falling is another important risk factor that warrants assessment as part of comprehensive falls prevention strategies for older adults [4]. Fear of falling, often expressed as concern about falling, can reduce confidence in daily activities and lead to activity restriction as a coping strategy, contributing to physical deconditioning and increased fall risk [6]. Greater concern about falling, as measured by a one-unit increase in the Falls Efficacy Scale International (FES-I) score, has been associated with a 5% increase in the odds of experiencing an injurious or recurrent fall [6]. Such behavioural adaptations may further impair balance and confidence, contributing to frailty, social isolation, and ultimately reduced overall quality of life [7].

In the Middle East and North Africa (MENA) region, falls pose a significant public health challenge shaped by regional factors such as limited access to geriatric care and sociocultural attitudes towards ageing and help-seeking behaviours [8–10]. MENA countries share regional policy frameworks, including those led by the World Health Organization (WHO) Eastern Mediterranean Regional Office and the League of Arab States, through which they often coordinate public health strategies, providing a relevant regional framework for synthesising evidence and identifying shared public health needs. They also share common languages, religious norms, extended family structures, and sociocultural perspectives on ageing and caregiving. However, MENA countries differ substantially in the pace of demographic transition, socioeconomic development, healthcare-system capacity, exposure to conflict, and the availability of geriatric services across low-, middle-, and high-income countries [11]. Alongside this heterogeneity, population ageing is accelerating across the region: the number of older adults in MENA is projected to triple by 2050, with the proportion of those aged ≥65 years expected to exceed 20% in six MENA countries and to increase 5-fold in others, including Qatar, Kuwait, and the United Arab Emirates [12]. This demographic transition underscores the need for regionally tailored fall prevention strategies [12,13].

Despite the growing burden of falls, comprehensive data on fall risk prevalence and fear of falling remain scarce. To date, no systematic review has synthesised this evidence among older adults in MENA, potentially constraining the development of effective policies and evidence-based public health interventions to reduce the fall burden.

In this systematic review, we address a critical knowledge gap by synthesising evidence on the prevalence of fall risk and fear of falling among adults aged ≥60 years across MENA countries. As the region undergoes rapid population ageing, with the fastest growth among those aged ≥60 years projected over the next three decades [12], we aim to inform evidence-based strategies to mitigate fall-related risks and improve quality of life among older adults in the MENA region.

## METHODS

### Protocol and registration

We formulated the research protocol *a priori* and prospectively registered it with Open Science Framework. Our systematic review was conducted in accordance with the Cochrane Collaboration Handbook [14] and complied with the PRISMA 2020 guidelines [15] (Table S1 in the **Online Supplementary Document**).

### Eligibility criteria

We formulated the eligibility criteria based on the Population, Intervention, Comparison, Outcome, Timing, and Setting framework [16]. We included studies that focused on our population of interest, older adults [17,18] aged ≥60 years, consistent with the United Nations definition [19], across the 20 MENA countries, which represent approximately 57 million individuals in this age group. Studies applying a ≥65-year threshold were also eligible, as this population falls within our definition of adults aged ≥60 years. Throughout this manuscript, we define older adults as individuals aged ≥60 years. Eligible studies included older adults living in community settings, residential care homes, and those attending outpatient or inpatient services, regardless of health status or medical conditions.

The classification of MENA countries is based on definitions established by the WHO, the Joint United Nations Programme on HIV/AIDS, the World Bank, and the Global Burden of Disease initiative [20–26]. This classification includes high-income countries (Bahrain, Kuwait, Oman, Qatar, Saudi Arabia, and the United Arab Emirates), upper-middle-income countries (Iraq, Jordan, and Libya), lower-middle-income countries (Algeria, Djibouti, Egypt, Lebanon, Morocco, Pakistan, Palestine, and Tunisia), and low-income countries (Sudan, Syria, and Yemen) [27].

We defined falls according to WHO guidelines as ‘an event resulting in a person coming to rest inadvertently on the ground, floor, or another lower level’, excluding falls caused by assault, self-harm, animals, and environmental hazards such as fire, water, and machinery [28]. We included studies reporting the prevalence of fall risk and fear of falling [4] encompassing both physical (*i.e.* balance, gait, functional mobility, and walking aid need) and psychological components (*i.e.* fear of falling measured as concerns about falling [4] and fall-related self-efficacy [29]). Fear of falling is defined as ‘an emotional response to a real or perceived threat to balance’ [30], while fall-related self-efficacy refers to an individual’s confidence to carry out daily activities without falling. Studies using both validated and non-validated assessment tools (*e.g.* single-item questionnaires) were eligible for inclusion in the review. We included all study designs except for case reports and case series. We placed no restrictions on the timing of data collection to ensure a comprehensive synthesis of fall risk assessment among older adults in the MENA region.

### Information sources and search strategy

We conducted extensive searches for grey and non-grey literature across PubMed/MEDLINE, Web of Science, Google Scholar, and Google in multiple languages (English, Arabic, and French) in November 2025, without applying time or language restrictions. Reference lists of included studies and relevant systematic reviews were also screened for potential additional inclusions. Our search used free-text terms and controlled vocabulary related to falls, older adults, and MENA countries (Boxes S1 and S2 in the **Online Supplementary Document**). An experienced librarian refined the search strategy to optimise database selection and search terms.

Two of three reviewers (AJ, SK, JA independently screened titles, abstracts, and full texts using Rayyan software [31]. An additional reviewer (KC) verified the excluded studies, and any conflicts were resolved through consensus, defined as mutual agreement reached through discussion; a third reviewer (KC) was consulted when consensus could not be achieved.

### Data extraction

Two of three reviewers (AJ, SK, JA) independently extracted data from all included studies using a standardised form, focusing on study characteristics (*i.e.* study design, sampling method, data collection period, sample size, and response rate), study setting, demographic information (*i.e.* population type, age, sex, and country of residence), assessment tools (*i.e.* tool name, validation status, and language of administration), and outcomes (*i.e.* mean score, standard deviation (SD), and prevalence estimates of higher fall risk and fear of falling, sample size, number of events). An additional reviewer (KC) verified the accuracy of the extracted data, with any conflicts resolved through consensus.

### Risk of bias assessment

Two of three reviewers (AJ, SK, JA) independently assessed the quality of each included study using the conservative ‘Risk of Bias Tool for Prevalence Studies’ developed by Hoy *et al.* [32]. Conflicts in the quality assessment were resolved through discussion; when consensus was not reached, a third reviewer (KC) was consulted. We evaluated each study across all assessment items, categorising each item as having a low or high risk of bias. A high risk of bias was assigned to studies that did not employ a validated tool to measure the reported outcomes of interest or where insufficient information precluded a judgment on an item [32]. This conservative approach aids in evaluating reporting biases within the included studies. Potential conflicts of interest were also investigated, following recommendations from the Cochrane Collaboration Handbook [14]. In line with COSMOS-E guidance [33], we did not calculate a summary quality score.

### Data synthesis

We synthesised data from primary studies that assessed fall risk using multifactorial and mobility-related assessment tools, as well as fear of falling (ie, concern about falling and fall-related self-efficacy). We conducted a quantitative synthesis using data from the most commonly used validated assessment tools. A meta-analysis was conducted for each assessment tool and outcome (prevalence and mean score), provided these were reported by at least three primary studies [14]. Studies employing validated tools that appeared in fewer than three included studies, as well as those employing non-validated tools (*e.g.* single-item questionnaires), were excluded from the meta-analysis (*i.e.* quantitative synthesis). These studies were included only in the qualitative synthesis, which involved a structured compilation of findings through narrative description, tabulated presentation of study characteristics, and risk-of-bias assessment.

Because the included instruments assess distinct domains related to fall risk, we conducted separate tool-specific meta-analyses to estimate the prevalence of older adults at higher fall risk and interpreted the findings according to each instrument’s assessed domain (multifactorial fall risk, balance performance, balance and gait performance, or functional mobility) and scoring criteria. Multifactorial fall risk was assessed using the Missouri Alliance for Home Care Fall Risk Assessment (MAHC-10) [34] and the Morse Fall Scale (MFS) [35]. A MAHC-10 score ≥4 and an MFS score >45 indicated higher fall risk. Balance performance was assessed using the Berg Balance Scale (BBS) [36], which is also indicative of the potential need for mobility support [37], with scores of 0–20 indicating high fall risk and a potential need for wheelchair assistance, and scores of 21–40 indicating moderate fall risk and a potential need for walking aids [38]. Balance and gait performance were assessed using the Tinetti Assessment Tool, with scores ≤18 indicating high fall risk and 19–23 indicating moderate fall risk [39]. Functional mobility, incorporating gait and dynamic balance performance [40,41], was assessed using the Timed Up and Go Test (TUGT) [42], with a time of ≥13.5 seconds indicating higher fall risk [43]. The World Guidelines for Falls Prevention [4] recommend TUGT as one of the assessment tools that are ‘useful and commonly used’ in the evaluation of both gait and balance.

Concern about falling and fall-related self-efficacy were treated as related but distinct constructs under the broader domain of fear of falling. Therefore, we conducted two separate meta-analyses to calculate the prevalence of fear of falling. The first meta-analysis focused on ‘fall-related self-efficacy’, the individual’s confidence in performing daily activities without falling, and assessed it using the Modified Falls Efficacy Scale (MFES), a 14-item instrument that includes four outdoor and community-based activities, such as walking on uneven surfaces or navigating crowded environments. A MFES score of <8 (or total score <112) indicates reduced confidence in performing daily activities without falling, whereas a score of 8–10 (or total score 112–140) indicates high confidence. The second meta-analysis addressed ‘concern about falling’, defined as emotional apprehension or worry about falling during physical and social activities, and assessed it using the FES-I [44] and its short version. These tools cover physical, social, and outdoor activities relevant to more active, community-dwelling older adults [45]. They are also specifically designed for cross-cultural translation [46]. A FES-I score of 28-64 indicates a high concern about falling, and 20-27 indicates a moderate concern. For the short FES-I, scores of 14-28 and 9-13 indicate high and moderate concern about falling, respectively. We also conducted a meta-analysis of mean scores for the FES-I and the original Falls Efficacy Scale (FES), a ten-item instrument focused mainly on basic indoor activities of daily living, such as dressing, bathing, or preparing simple meals [45]. The original FES was mainly designed for frail or homebound older adults.

### Data analysis

We conducted meta-analyses to estimate pooled weighted-average prevalence and mean scores using a random-effects model. We included only studies that used standard tools. We used the ‘metaprop’ and ‘metamean’ functions in *R*, version 4.0.3 (R Core Team, Vienna, Austria).

To assess between-study heterogeneity, we employed Cochrane’s Q statistic and the *I*^2^ statistic, which quantifies the proportion of variability attributable to true differences in prevalence or mean estimates rather than chance. Substantial heterogeneity was defined as *I*^2 ^> 50% [47]. We conducted random-effects meta-regressions to explore heterogeneity related to study methodology (*e.g.* assessment tool, sample size, response rate, sampling method, year of data collection), social determinants (*e.g.* age, sex, country, and World Bank income classification), and population types. Univariate meta-regression was performed using the ‘metareg’ function from the *R* meta package, applying logit transformation to compute odds ratios (ORs) and quantify associations between sources of heterogeneity and effect size. Given the limited number of studies/data points available across several covariate categories and to avoid overfitting, meta-regression analyses were restricted to univariate models.

We estimated missing years of data collection by adjusting the year of publication based on the median difference between the year of publication and the year of data collection. We calculated this median difference from studies that provided complete information.

Sensitivity analyses were conducted by repeating the meta-regression for year of data collection after excluding data points from studies with imputed years. Additionally, to examine whether methodological quality was associated with prevalence estimates, we conducted meta-regression analyses for individual items of the ‘Risk of Bias Tool for Prevalence Studies’ [32], comparing estimates according to low *vs.* high risk of bias. Items with no variation in risk-of-bias ratings were not evaluated.

We employed Doi plots, displaying normal-quantile (Z-score on the y-axis) against the logit-transformed prevalence (x-axis), and the Luis Furuya–Kanamori (LFK) index, to assess publication bias by visualising asymmetry and quantifying potential asymmetry in study effects [48]. An LFK index less than −1 or greater than +1 indicated publication bias. We opted not to use funnel plots and Egger's regression for publication bias assessment because these approaches have methodological limitations in meta-analyses of proportions, where funnel-plot asymmetry may arise even in the absence of publication bias [49]. The Doi plot and LFK index were therefore used as alternative graphical and quantitative approaches to assessing asymmetry [48].

### Certainty of evidence assessment

We applied the GRADE approach to evaluate the certainty of the synthesised evidence in the systematic review. This appraisal involved discussions regarding the validity and reliability of our estimates, considering study-level quality assessment, reporting biases, precision of estimates, consistency of primary study results, and the directness of the evidence addressing the research question [50]. Substantial between-study heterogeneity was considered under the inconsistency domain. We assessed imprecision separately based on the sample sizes contributing to each pooled estimate and the estimate's uncertainty, while the methodological quality of the contributing studies informed the risk-of-bias domain.

## RESULTS

### Study selection and characteristics

We identified 12,399 records, from which 109 studies were included in the systematic review, providing data on 213,877 older adults across ten countries (**Figure 1**; Table S2 in the **Online Supplementary Document**). These included studies from Egypt (n = 44) [51–94], Iraq (n = 2) [95,96], Jordan (n = 2) [97,98], Lebanon (n = 1) [99], Morocco (n = 1) [100], Pakistan (n = 20) [101-120], Palestine (n = 3) [121–123], Saudi Arabia (n = 32) [124–155], Tunisia (n = 2) [156,157], and the United Arab Emirates (n = 2) [158,159]. Participants in the included studies ranged in age from 60 to 110 years, with reported mean ages of 63.1 (SD = 2.5) and 77.2 (SD = 4.6) years. Of these studies, 43 reported the prevalence of older adults at higher fall risk, while 58 reported mean fall-risk scores. Additionally, 27 studies assessed fear of falling, with 22 reporting mean fear-of-falling scores.

**Figure 1:**
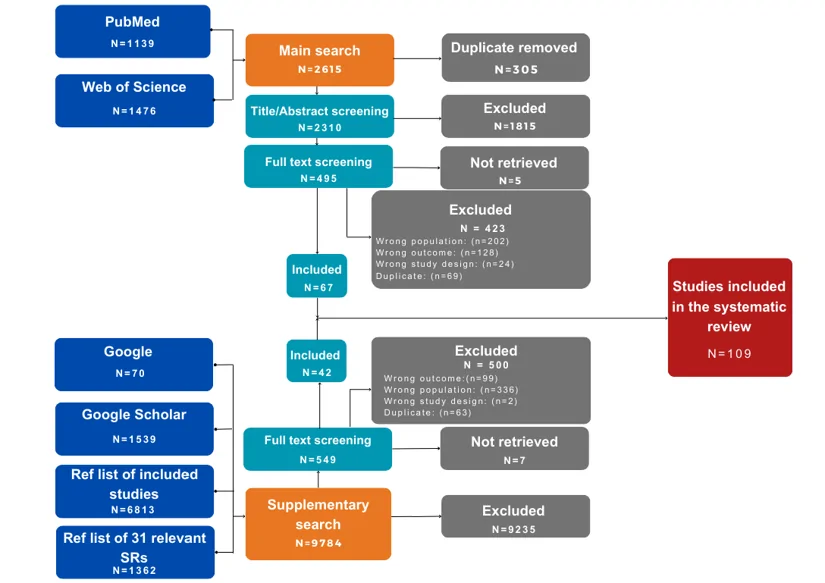
Primary study selection flow diagram.

### Assessment of quality

Overall, the studies demonstrated good internal validity (Figure S1 and Table S3 in the **Online Supplementary Document**). All included studies consistently used the same data-collection method for all participants, used appropriate time frames to measure fall risk and fear of falling among older adults, used accurate numerators and denominators to estimate prevalence, and collected data directly from participants or their medical records. Although studies intended to assess unintentional falls, only 57% provided a clear definition of the outcome. Studies assessed fall risk and fear of falling using validated tools in 99% and 76% of cases, respectively. External validity was harder to evaluate because 87% of studies did not report response rates. However, 96% of studies used population samples closely representative of the target population, although 82% were not representative of the country’s population. No potential conflicts of interest were disclosed in the studies that reported on this aspect.

### Fall risk assessments

Studies used a range of multi-factorial fall risk assessment tools, including the MAHC-10 [34] (n = 4) [125,127,128,137] and the MFS [35] (n = 5) [52,53,80,127,129], Hendrich II Fall Risk model (n = 3) [51,52,108], Johns Hopkins Fall Risk Assessment Tool (n = 1) [52], Schmid Fall Risk Assessment (n = 3) [52,71,159], Elderly Fall Screening Test (n = 1) [105], Fall Risk for Older People- Community Setting (n = 2) [56,57], and Fall Risk Assessment Scale (n = 3) [63,81,82]. One study [130] reported fall risk prevalence without specifying the assessment tool used. The pooled prevalence of older adults at higher fall risk was 54.2% on the MAHC-10 (n datapoints = 6; n = 1008; 95% confidence interval (CI) = 35.1, 72.8; *I*^2^ = 97%) and 33.4% on the MFS (n datapoints = 7; n = 1391; 95% CI = 18.4, 53.8; *I*^2^ = 97%).

The included studies measured balance performance primarily using the BBS (n = 33) [36,51,54,57,59,62,67,71,74,77-79,84,85,95,99,102,106,108,109,113–116,118,119,143,144,147–150,152] and balance and gait performance using the Tinetti Assessment Tool (n = 5) [66,89,100,124,152]. The pooled mean BBS score across studies was 40.1 (n datapoints = 41; n = 2439; 95% CI = 36.9, 43.2; *I*^2^ = 100%) (Table S4 in the **Online Supplementary Document**). Subgroup meta-analysis showed that pooled mean scores differed significantly by population type, while no significant differences were observed by sex. The Tinetti Assessment Tool yielded a pooled mean score of 20.0 (n datapoints = 9; n = 239; 95% CI = 18.7, 21.4; *I*^2^ = 83.0%), which falls within the range of 19–23, indicating a moderate fall risk [39]. Subgroup meta-analysis also showed significant differences by population type.

Among older adults in the MENA region, the pooled prevalence of those at high risk of falls (BBS score 0–20) was 16.6% (n datapoints = 12; n = 1677; 95% CI = 7.9, 31.4; *I*^2^ = 95.0%), indicating a potential need for wheelchair assistance. Additionally, 22.9% (n datapoints = 11; n = 1600; 95% CI = 14.0, 35.1; *I*^2^ = 94.0%) were at a moderate risk (BBS score 21–40), indicating potential need for walking aids (**Figure 2**). The overall pooled prevalence of older adults at higher fall risk (BBS score = 0–40) was 41.7% (n datapoints = 11; n = 1600; 95% CI = 27.6, 57.2; *I*^2^ = 95.8%).

**Figure 2:**
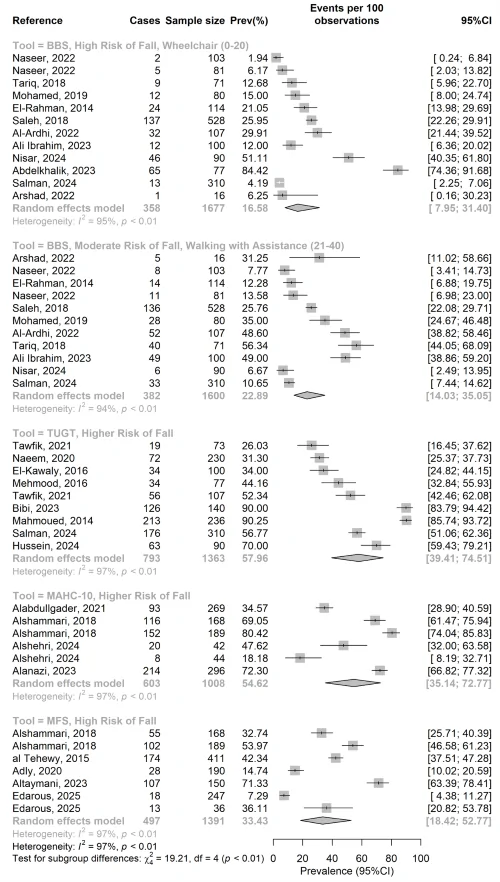
Meta-analysis of the proportion of older adults at higher fall risk based on balance performance, functional mobility, and multifactorial fall-risk assessments. BBS – Berg Balance Scale, CI – confidence interval, MAHC-10 – Missouri Alliance for Home Care Fall Risk Assessment, MFS – Morse Fall Scale, TUGT – Timed Up and Go Test.

The Five Times Sit-to-Stand Test (n = 3) [126,131,145] was used as a lower-limb functional performance measure related to fall risk. Functional mobility, incorporating gait and dynamic balance performance, was evaluated using the TUGT [42] (n = 20) [55,58,59,64,65,68,70,71,74,75,100,102,103,105,121,122,124,126,130,158], with a pooled mean time of 15.4 seconds (n datapoints = 66; n = 4204; 95% CI = 14.2, 16.6; *I*^2^ = 98%). A time of ≥13.5 seconds indicated a higher risk of falling [43]. Subgroup meta-analysis showed significant differences in pooled mean scores by population type (Table S4 in the **Online Supplementary Document**), but not by sex. In the MENA region, the pooled prevalence of older adults at higher fall risk based on the TUGT was 58.0% (n datapoints = 9; n = 1363; 95% CI = 39.4, 74.5; *I*^2^ = 97%).

The univariate meta-regression analyses found no statistically significant associations between higher fall risk prevalence and population type, sex, World Bank income levels, assessment tools, age cutoff, sample size, response rate, sampling methodology, or year of data collection (Table S5 in the **Online Supplementary Document**). Sensitivity analyses excluding studies with imputed years of data collection yielded similar findings. Year of data collection remained not significantly associated with the prevalence of higher fall risk (k = 29; *β* = −0.022; 95% CI = −0.135, 0.091; *P* = 0.703). Risk-of-bias meta-regression analyses showed no statistically significant associations between the risk-of-bias items and prevalence estimates of higher fall risk (all *P* > 0.05; Table S6 in the **Online Supplementary Document**). Items 5, 7, 8, 9, and 10 from the ‘Risk of Bias Tool for Prevalence Studies’ [32] had only a low risk of bias rating; therefore, meta-regression was not feasible for these items.

### Fear of falling

Three [83,112,155] included primary studies reporting fear of falling used the original FES [45], a ten-item instrument, focused mainly on basic indoor activities of daily living, such as dressing, bathing, or preparing simple meals. Most studies used validated tools measuring concern about falling, including the FES-I (n = 15) [61,86,88,90,98,108,111,114,117,118,133,143,144,146,147] and short FES-I (n = 1) [54], and tools that measure fall related self-efficacy, MFES (n = 8) [65,69,72–74,76,110,151]. An additional study [145] used the Iconographical Falls Efficacy Scale; two studies used the visual analogue scale [59,158], five studies assessed fear of falling using single-item or study-specific questionnaires [55,63,73,97,126], and three other studies [60,96,110] reported assessing fear of falling, but did not specify the assessment tool used. The pooled mean score across studies on the FES-I was 33.6 (n datapoints = 17; n = 1842; 95% CI = 29.4, 37.8; *I*^2^ = 99%) and on the FES was 23.9 (n datapoints = 4; n = 766; 95% CI = 12.9, 34.9; *I*^2^ = 99%) (Figure S2 in the **Online Supplementary Document**).

Across studies, 82.7% of older adults (n datapoints = 7; n = 816; 95% CI = 60.4, 93.8; *I*^2^ = 92%) expressed moderate to high concern about falling during physical and social activities (FES-I) and 67.1% (n datapoints = 9; n = 1573; 95% CI = 44.9, 83.6; *I*^2^ = 97%) expressed reduced confidence in carrying out daily activities without falling (MFES) (**Figure 3**).

**Figure 3:**
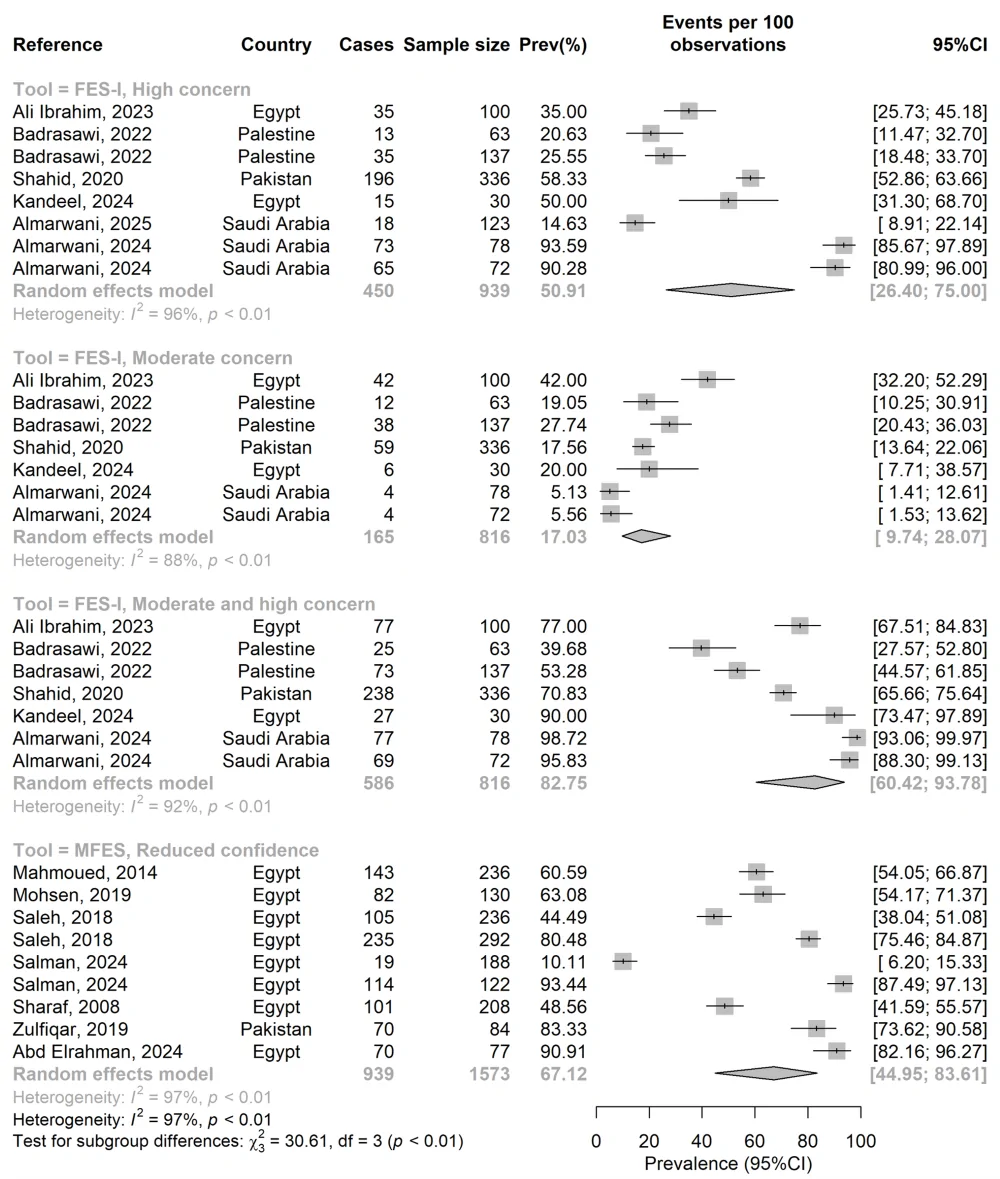
Meta-analysis of the proportion of older adults with fear of falling, categorised by level of concern about falling and fall-related self-efficacy. CI – confidence interval, MFES – Modified Falls Efficacy Scale, FES-I – Falls Efficacy Scale International.

The univariate meta-regressions showed no statistically significant associations between the prevalence measures of fear of falling and the assessment tools, population type, sex, World Bank income levels, age cutoff, sample size, response rate, sampling methodology, and year of data collection (Table S7 in the **Online Supplementary Document**). Sensitivity analysis excluding studies with an imputed year of data collection yielded similar findings, with no statistically significant association between year of data collection and the prevalence measures of fear of falling (k = 20; *β* = −0.130, 95% CI = −0.329, 0.068; *P* = 0.199). Risk-of-bias meta-regression analyses showed no statistically significant associations between the risk-of-bias items and prevalence estimates of fear of falling (all *P* > 0.05) (Table S8 in the **Online Supplementary Document**). Items 2, 5, 7, 8, 9, and 10 from the ‘Risk of Bias Tool for Prevalence Studies’ [32] had only a low risk of bias rating; therefore, meta-regression was not feasible for these items.

### Reporting bias and certainty of the evidence

We detected no evidence of publication bias for fear of falling (LFK index = −0.14), whereas we found minor asymmetry for fall risk (LFK index = −1.37) (Figure S3 in the **Online Supplementary Document**). However, the absence of studies from ten countries and the small number of nationwide studies may limit the generalisability of the regional pooled prevalence estimates. Despite these limitations, the generally good methodological quality of the included studies supported confidence in the evidence, while the sample sizes contributing to the pooled estimates reduced concerns about imprecision. Nevertheless, substantial between-study heterogeneity indicated some inconsistency and reduced confidence in the pooled estimates, resulting in a moderate certainty rating for the evidence.

## DISCUSSION

We found a substantial prevalence of higher fall risk and fear of falling among older adults across several MENA countries, underscoring the urgent need for context-specific fall prevention interventions in the region. We found that 41.7% of older adults were at higher risk of falls based on balance assessments, while 58.0% were at higher risk based on functional mobility assessments. Multifactorial fall-risk assessments identified pooled prevalence estimates of higher fall risk ranging from 33.4% to 54.2%. Furthermore, 82.7% of older adults reported moderate to high concern about falling during physical and social activities, while 67.1% expressed reduced confidence in carrying out daily activities without falling, emphasising the substantial psychological burden related to fear of falling alongside physical factors related to fall risk. These findings should be interpreted according to the specific domains assessed: balance assessments evaluate balance performance, functional mobility assessments include gait and dynamic balance performance, and multifactorial fall-risk tools include a broader range of clinical risk factors. Although these domains are related to fall risk, they are not interchangeable.

These findings highlight the importance of multi-component screening approaches and interventions that address both physical and psychological fall-related risk factors. Region-specific approaches, informed by shared characteristics such as coordinated policy frameworks, common languages, sociocultural norms, and caregiving structures, are necessary to support effective implementation within the MENA context. However, recognising common regional factors does not imply a uniform approach across all MENA countries. Implementation contexts vary substantially because of differences in healthcare infrastructure, resource availability, and fall risk profiles. Therefore, prevention strategies should be coordinated regionally while adapting to national contexts to improve feasibility, acceptability, and long-term sustainability.

Although we found no statistically significant differences in fall-risk prevalence by income level, this finding should be interpreted cautiously given the limited number of countries with available data. Socioeconomic inequalities and variations in healthcare infrastructure across the MENA region are likely to contribute to broader public health challenges, particularly those related to population ageing, long-term care needs, and social welfare coverage [160]. These factors may also shape how the region implements and scales fall prevention programmes. For example, higher-income countries may be better positioned to integrate telehealth services and smart home technologies with structured exercise programmes as part of community-based fall prevention strategies [4]. However, resource constraints and the need for cost-effective allocation may make such approaches infeasible or inappropriate in low- and middle-income settings. As the World Falls Guidelines [4] highlight, low- and middle-income countries often face structural challenges, including shortages in the geriatric workforce, limited access to rehabilitation services, and underdeveloped preventive infrastructure, which go beyond country-level differences in prevalence. In such contexts, fall prevention efforts may prioritise community health worker-led interventions and low-cost, scalable screening approaches [161]. Additionally, the feasibility of implementing standardised fall-risk assessment tools in routine primary care may vary across MENA settings depending on workforce capacity, staff training, and available healthcare resources, and implementation may therefore require adaptation to local service contexts [4]. Across all settings, addressing physical factors such as balance and functional mobility limitations, together with psychological factors such as fear of falling and broader multifactorial fall risk, remains essential. Overall, these findings support the need for fall prevention strategies in the MENA region that are coordinated at the regional level while being adapted to the national context, healthcare capacity, and population needs.

We found that a substantial proportion of older adults in MENA countries with available data were classified as high risk for falls based on balance and functional mobility assessments. The absence of comparable global estimates for older adults at high risk of falling precluded direct regional comparisons. Nevertheless, the prevalence of fear of falling we observed appears higher than estimates reported from Turkey (44.6%) [162] and the UK (18.4%) [163], although differences in assessment tools, study populations, and definitions should be considered when making such comparisons. Self-reported fear of falling, including concern about falling and fall-related self-efficacy, may be influenced by sociocultural context, independent of objective fall risk. Cultural perceptions of ageing, frailty, and dependence, as well as family support and caregiving structures, may shape how older adults perceive and report fear of falling and may partly contribute to differences in reported prevalence between studies and countries [164,165]. Fear of falling often leads to reduced physical activity, which may exacerbate frailty and accelerate muscle loss, contributing to sarcopenia. This self-reinforcing cycle increases the risk of social isolation, mental health problems, and subsequent fall risk [7]. Addressing the psychological dimension of fall risk is therefore critical [166], as the interplay between fear of falling, sarcopenia, physical health, and mobility highlights the multifactorial nature of falls. Our findings underscore the need for holistic fall prevention interventions in MENA that integrate balance and gait training (*e.g.* the Otago Exercise Program), psychological interventions (*e.g.* cognitive behavioural therapy for fear of falling), and routine fall risk screening in primary care to reduce fall risk and support healthy ageing [4].

Our study has several limitations, particularly related to the availability of country-specific data. Although we detected no publication bias for fear of falling, we observed minor asymmetry for fall risk using the Doi plot and LFK index, and substantial gaps in data coverage persist across the MENA region. Specifically, no data were available for Bahrain, Kuwait, Oman, and Qatar (high-income countries); Libya (upper-middle-income country); Algeria and Djibouti (lower-middle-income countries); and Sudan, Syria, and Yemen (low-income countries). This data gap limited subgroup analyses (*e.g.* by sex and age group) and may have affected the accuracy of the estimated prevalence of higher fall risk and fear of falling. The lack of data from ten countries may limit the generalisability of our findings to the entire MENA region. The reasons for these evidence gaps are likely to differ across countries, ranging from a lack of studies specifically addressing fall-related risk factors among older adults in some settings to conflict and constraints in research and healthcare systems in others [11,167]. Consequently, the countries without available data may differ systematically from those represented in the review, potentially influencing the regional pooled estimates.

In addition, several tool-specific pooled estimates were based on relatively few studies, despite generally adequate overall sample sizes, and should therefore be interpreted cautiously. More broadly, although pooling data across MENA provides a useful regional summary of common patterns, it may also mask important between-country differences in demographic, socioeconomic, and healthcare contexts. Therefore, the estimated pooled prevalence and pooled mean scores should be interpreted as regional averages across heterogeneous populations rather than precise country-level estimates that apply uniformly to all MENA countries. While the available studies offer valuable insights into regional fall-risk patterns, limited data availability may constrain the development of effective prevention strategies and efforts to address health disparities across the region [1]. Strengthening data collection systems, particularly in countries that lack fall-related data, is crucial to inform public health policy and guide effective interventions. Enhanced data collection and surveillance efforts, such as national health surveys and community-based fall registries, are essential for identifying at-risk populations and guiding evidence-based fall prevention initiatives [168].

The combined effects of ageing, reduced mobility, and mental health challenges affect not only physical health but also social well-being and quality of life [169]. Addressing these complex health challenges is particularly difficult in the MENA region, where, despite shared cultural characteristics, socioeconomic disparities between high-, middle-, and low-income countries create significant barriers to implementing region-wide fall prevention strategies. These differences in resources and healthcare capacity directly affect each country's ability to address fall risk and protect older adults' well-being.

The use of ≥60 years to define older adults was consistent with the United Nations definition [19] and with the age threshold applied in the main regional and global evidence [8,9] used for comparison in this study. Studies using a ≥65-year threshold were also eligible. Although our meta-regression found no statistically significant association between age definition and prevalence estimates, differences in age thresholds should still be considered when comparing findings across studies, as some geriatric studies define older adults as individuals aged ≥65 years.

Our meta-regression analysis revealed no statistically significant associations between the prevalence of higher fall risk or fear of falling and population type, sex, or country-income level. These non-significant findings should not be interpreted as evidence of equivalence between subgroups, as the meta-regression analyses may have had reduced statistical power to detect differences. In contrast, subgroup meta-analysis of mean scores from tools assessing balance performance and functional mobility revealed significant differences by population type. These findings suggest that fall risk factors are prevalent across MENA subpopulations, while mobility performance may vary by population type. When interpreting the pooled estimates, it should be considered that they represent averages across populations with different underlying health status, functional ability, and comorbidity. Consequently, universal fall-risk screening may be warranted [170], complemented by context-specific interventions tailored to population needs. Population-wide strategies therefore help improve healthy ageing while ensuring that higher-risk groups receive appropriate targeted support.

Many included studies had risks of non-response and selection biases, which are common in cross-sectional studies using questionnaires [171]. The lack of nationwide data limits the national representativeness of the findings, warranting caution when generalising across various contexts. Our meta-analysis also identified substantial heterogeneity, which is commonly observed in meta-analyses of prevalence estimates [172]. This heterogeneity likely stems from variations in study methodologies, sample sizes, and settings. Previous meta-analyses have similarly reported high heterogeneity due to variations in study methodologies and population characteristics [173]. We applied a random-effects model to ensure pooled estimates account for these variations. Considering these challenges, we also conducted meta-regression analyses to explore the sources of heterogeneity. However, the limitations in data representativeness persist. Therefore, the calculated estimates should be interpreted as regional weighted averages summarising heterogeneous study populations and settings, indicating a broader regional pattern rather than definitive figures applicable uniformly across the MENA region. Although variability exists across studies, the consistently high prevalence of fall risk and fear of falling underscores the need for routine fall-risk screening and targeted interventions in geriatric care across MENA countries.

We address a critical global health issue associated with population ageing, focusing on the MENA region, which remains underrepresented in fall-related research. This review provides the first pooled regional prevalence estimates of key physical and psychological fall-related risk factors in MENA, emphasising both their substantial magnitude and the considerable variability in assessment approaches across countries. By synthesising region-specific data, this study provides evidence to inform targeted, large-scale public health policies and strategies to support healthy ageing in MENA populations. We acknowledge the heterogeneity of fall risk assessment tools used across the included studies. First, we retained studies using non-validated instruments in the qualitative synthesis to capture the range of assessment approaches used across the region; however, interpret their findings cautiously, as the lack of validation may affect reliability and comparability. These studies did not contribute to the pooled estimates. Second, we also included studies using different validated tools. As previously noted [5], these tools are often developed for specific settings such as hospitals, long-term care facilities, or community-based populations and differ in structure and purpose. They also assess related but distinct dimensions of fall-related vulnerability; therefore, pooled estimates derived from different instruments should not be interpreted as directly comparable measures of the same construct. Differences between tool-specific estimates may reflect variation in the domains assessed, scoring thresholds, and intended clinical settings. Their inclusion in this review and corresponding meta-analyses reflects real-world usage across the region.

Among these tools, only MAHC-10 and MFS were used in a sufficient number of studies to permit meta-analyses. While MAHC-10 is primarily designed for home care settings and MFS is more commonly applied in hospital settings, both are validated tools for assessing multifactorial fall risk. These tools incorporate several recommended domains for evaluating fall risk, including fall history, medication use, cognitive status, and gait or balance [4]. Our findings underscore the need to adopt standardised, context-appropriate fall risk assessment tools for use in community and primary care settings across MENA to support consistent screening, comparable data, and effective fall prevention planning.

In addition to multifactorial risk assessments, this review incorporates validated tools that assess key physical and psychological fall-related risk factors. These include the BBS, which assesses balance performance, and the TUGT, which assesses functional mobility incorporating gait and dynamic balance [174]. Additionally, it incorporates the FES-I tool and its short version, as well as the MFES, which are globally acknowledged tools for assessing the psychological dimensions of falls and have demonstrated applicability across diverse cultural settings [175]. The FES-I measures concern about falling, while the MFES evaluates confidence in performing daily activities without falling. Both tools are conceptually derived from the original FES. While the original FES was primarily developed for frail or homebound older adults, the MFES and FES-I include a broader range of indoor, outdoor, and social activities, making them applicable to more diverse older adult populations. Moreover, although the World Falls Guidelines recommend the terminology 'concern about falling,' we use 'fear of falling' throughout this review to remain consistent with the terminology employed in the included primary studies, which assessed not only concern about falling but also fall-related self-efficacy [4]. Our systematic review recognises the psychological consequences of falls, such as fear of falling, which can lead to reduced self-confidence, activity restriction, social isolation, and an increased risk of physical frailty [176]. Integrating physical and psychological assessments therefore enables a more comprehensive evaluation of fall risk among older adults in the MENA region.

## CONCLUSIONS

We highlight the substantial prevalence of higher fall risk and fear of falling among older adults in the MENA countries represented in the available evidence. Given the substantial heterogeneity in study populations, assessment methods, and geographical coverage, the pooled estimates should be interpreted as regional averages across heterogeneous populations rather than as estimates applicable uniformly across all MENA countries. These findings support fall prevention strategies that address both physical factors (balance and functional mobility) and psychological factors (fear of falling). Strengthening data collection and surveillance systems across MENA, particularly through standardised and nationally representative studies in underrepresented countries, is crucial to inform evidence-based policy, enhance geriatric care, and integrate fall prevention into national healthcare frameworks. A comprehensive, context-specific approach may help reduce fall-related morbidity and support healthy longevity in this rapidly ageing region.

## Additional material


Online Supplementary Document

## Data Availability

**Data availability:** All data generated or analysed during this study are included in this published article and its supplementary information files. Further inquiries can be directed to the corresponding author.
