# Supplementary material for: Prevalence of fall risk and fear of falling among older adults in the Middle East and North Africa: a systematic review and meta-analysis
Source: J Glob Health. 2026 Oct 30;16:04397. doi: 10.7189/jogh.16.04397 (PMC13721842; doi:10.7189/jogh.16.04397)
Supplement: Supplementary file 1 [file jogh-16-04397-s001.pdf]

Supplement to: Chaabna K, Jithesh A, Khawaja S, Aboughanem J, Mamtani R, Cheema S. Prevalence of fall risk and fear of falling among older adults in the Middle East and North Africa: a systematic review andmeta-analysis. J Glob Health. 2026;16:04397.

Table S1: Prisma 2020 checklist

| Section and Topic | Item # | Checklist item | Location where item is reported |
|---|---|---|---|
| TITLE | | | |
| Title | 1 | Identify the report as a systematic review. | Title |
| ABSTRACT | | | |
| Abstract | 2 | See the PRISMA 2020 for Abstracts checklist. | Abstract |
| INTRODUCTION | | | |
| Rationale | 3 | Describe the rationale for the review in the context of existing knowledge. | Introduction |
| Objectives | 4 | Provide an explicit statement of the objective(s) or question(s) the review addresses. | Introduction |
| METHODS | | | |
| Eligibility criteria | 5 | Specify the inclusion and exclusion criteria for the review and how studies were grouped for the syntheses. | Eligibility criteria |
| Information sources | 6 | Specify all databases, registers, websites, organisations, reference lists and other sources searched or consulted to identify studies. Specify the date when each source was last searched or consulted. | Information sources and search strategy |
| Search strategy | 7 | Present the full search strategies for all databases, registers and websites, including any filters and limits used. | Information sources and search strategy and Box S1 and S2 |
| Selection process | 8 | Specify the methods used to decide whether a study met the inclusion criteria of the review, including how many reviewers screened each record and each report retrieved, whether they worked independently, and if applicable, details of automation tools used in the process. | Information sources and search strategy |
| Data collection process | 9 | Specify the methods used to collect data from reports, including how many reviewers collected data from each report, whether they worked independently, any processes for obtaining or confirming data from study investigators, and if applicable, details of automation tools used in the process. | Data extraction |
| Data items | 10a | List and define all outcomes for which data were sought. Specify whether all results that were compatible with each outcome domain in each study were sought (e.g. for all measures, time points, analyses), and if not, the methods used to decide which results to collect. | Data extraction |
| | 10b | List and define all other variables for which data were sought (e.g. participant and intervention characteristics, funding sources). Describe any assumptions made about any missing or unclear information. | Data extraction |
| Study risk of bias | 11 | Specify the methods used to assess risk of bias in the included studies, including details of the tool(s) used, how many reviewers assessed each study and whether they worked independently, and if applicable, details of automation tools used in the process. | Risk of bias assessment |

| Section and Topic | Item # | Checklist item | Location where item is reported |
|---|---|---|---|
| assessment | | | |
| Effect measures | 12 | Specify for each outcome the effect measure(s) (e.g. risk ratio, mean difference) used in the synthesis or presentation of results. | Data synthesis |
| Synthesis methods | 13a | Describe the processes used to decide which studies were eligible for each synthesis (e.g. tabulating the study intervention characteristics and comparing against the planned groups for each synthesis (item #5)). | Data synthesis |
| | 13b | Describe any methods required to prepare the data for presentation or synthesis, such as handling of missing summary statistics, or data conversions. | Data synthesis |
| | 13c | Describe any methods used to tabulate or visually display results of individual studies and syntheses. | Data synthesis |
| | 13d | Describe any methods used to synthesize results and provide a rationale for the choice(s). If meta-analysis was performed, describe the model(s), method(s) to identify the presence and extent of statistical heterogeneity, and software package(s) used. | Data synthesis |
| | 13e | Describe any methods used to explore possible causes of heterogeneity among study results (e.g. subgroup analysis, meta-regression). | Data synthesis |
| | 13f | Describe any sensitivity analyses conducted to assess robustness of the synthesized results. | Data synthesis |
| Reporting bias assessment | 14 | Describe any methods used to assess risk of bias due to missing results in a synthesis (arising from reporting biases). | Data synthesis |
| Certainty assessment | 15 | Describe any methods used to assess certainty (or confidence) in the body of evidence for an outcome. | Certainty of evidence assessment |
| RESULTS | | | |
| Study selection | 16a | Describe the results of the search and selection process, from the number of records identified in the search to the number of studies included in the review, ideally using a flow diagram. | Study selection and characteristics |
| | 16b | Cite studies that might appear to meet the inclusion criteria, but which were excluded, and explain why they were excluded. | Study selection and characteristics |
| Study characteristics | 17 | Cite each included study and present its characteristics. | Study selection and characteristics |
| Risk of bias in studies | 18 | Present assessments of risk of bias for each included study. | Assessment of quality and Figure S1 and Table S3 |
| Results of | 19 | For all outcomes, present, for each study: (a) summary statistics for each group (where appropriate) and (b) an effect estimate and its | Results |

| Section and Topic | Item # | Checklist item | Location where item is reported |
|---|---|---|---|
| individual studies | | precision (e.g. confidence/credible interval), ideally using structured tables or plots. | |
| Results of syntheses | 20a | For each synthesis, briefly summarise the characteristics and risk of bias among contributing studies. | Results |
| | 20b | Present results of all statistical syntheses conducted. If meta-analysis was done, present for each the summary estimate and its precision (e.g. confidence/credible interval) and measures of statistical heterogeneity. If comparing groups, describe the direction of the effect. | Results |
| | 20c | Present results of all investigations of possible causes of heterogeneity among study results. | Results |
| | 20d | Present results of all sensitivity analyses conducted to assess the robustness of the synthesized results. | Results |
| Reporting biases | 21 | Present assessments of risk of bias due to missing results (arising from reporting biases) for each synthesis assessed. | Reporting bias and certainty of the evidence |
| Certainty of evidence | 22 | Present assessments of certainty (or confidence) in the body of evidence for each outcome assessed. | Reporting bias and certainty of the evidence |
| DISCUSSION | | | |
| Discussion | 23a | Provide a general interpretation of the results in the context of other evidence. | Discussion |
| | 23b | Discuss any limitations of the evidence included in the review. | Discussion |
| | 23c | Discuss any limitations of the review processes used. | Discussion |
| | 23d | Discuss implications of the results for practice, policy, and future research. | Discussion |
| OTHER INFORMATION | | | |
| Registration and protocol | 24a | Provide registration information for the review, including register name and registration number, or state that the review was not registered. | Methods-Protocol and registration |
| | 24b | Indicate where the review protocol can be accessed, or state that a protocol was not prepared. | Methods-Protocol and registration |
| | 24c | Describe and explain any amendments to information provided at registration or in the protocol. | NA |
| Support | 25 | Describe sources of financial or non-financial support for the review, and the role of the funders or sponsors in the review. | Funding |
| Competing interests | 26 | Declare any competing interests of review authors. | Competing interest |
| Availability of data, code and | 27 | Report which of the following are publicly available and where they can be found: template data collection forms; data extracted from included studies; data used for all analyses; analytic code; any other materials used in the review. | Availability of data and |

| Section and Topic | Item # | Checklist item | Location where item is reported |
| --- | --- | --- | --- |
| other materials | | | materials section in the manuscript |

Box S1: Search Strategy—Main search

| Electronic databases | Date of search |
|---|---|
| PubMed/Medline: 1139 | $13^{th}$ November 2025 |
| ("aged"[MeSH Terms] OR "aged"[TEXT WORD] OR "elderly*"[TEXT WORD] OR "older population*"[TEXT WORD] OR geriatric*[TEXT WORD] or “older adult*”[TEXT WORD] OR "Falls in older adult*"[TEXT WORD])<br>AND<br>("accidental falls"[MeSH Terms] OR ("accidental"[TEXT WORD] AND "falls"[TEXT WORD]) OR "accidental falls"[TEXT WORD] OR "falling"[TEXT WORD] OR "falls"[TEXT WORD] OR "fallings"[TEXT WORD] or "accidental slip*"[TEXT WORD] OR “unintentional fall*”[TEXT WORD] or “loss of balance”[TEXT WORD])<br>AND<br>("Qatar"[Mesh] OR "Bahrain"[Mesh] OR "Oman"[Mesh] OR "Saudi Arabia"[Mesh] OR "Kuwait"[Mesh] OR "United Arab Emirates"[Mesh] OR "Yemen"[Mesh] OR "Egypt"[Mesh] OR "Jordan"[Mesh] OR "Lebanon"[Mesh] OR "Syria"[Mesh] OR "Iraq"[Mesh] OR "Algeria"[Mesh] OR "Libya"[Mesh] OR "Morocco"[Mesh] OR "Tunisia"[Mesh] OR "Djibouti"[Mesh] OR "Sudan"[Mesh] OR "South Sudan"[Mesh] OR "Pakistan"[Mesh] OR "Africa, Northern"[Mesh] OR "Africa, Eastern"[Mesh] OR "middle east"[Mesh] OR "Arabs"[Mesh] OR "UAE"[Title/Abstract] OR "U.A.E"[Title/Abstract] OR "Emirat*"[Title/Abstract] OR "United Arab Emirates"[Title/Abstract] OR "Qatar*"[Title/Abstract] OR "Oman*"[Title/Abstract] OR "Saudi Arabia*"[Title/Abstract] OR "Saudi*"[Title/Abstract] OR "Kuwait*"[Title/Abstract] OR "Bahrain*"[Title/Abstract] OR "Yemen*"[Title/Abstract] OR "Egypt*"[Title/Abstract] OR "Jordan*"[Title/Abstract] OR "Leban*"[Title/Abstract] OR "Syria*"[Title/Abstract] OR "Iraq*"[Title/Abstract] OR "West Bank*"[Title/Abstract] OR "Gaza*"[Title/Abstract] OR "Palestin*"[Title/Abstract] OR "Algeria*"[Title/Abstract] OR "Libya*"[Title/Abstract] OR "Morocc*"[Title/Abstract] OR "Tunis*"[Title/Abstract] OR "Djibouti*"[Title/Abstract] OR "Sudan*"[Title/Abstract] OR "South Sudan*"[Title/Abstract] OR "Pakistan*"[Title/Abstract] OR "North Africa*"[Title/Abstract] OR "North-Africa*"[Title/Abstract] OR ("Africa"[Title/Abstract] AND "Northern"[Title/Abstract]) OR "Northern Africa"[Title/Abstract] OR "East Africa"[Title/Abstract] OR ("Africa"[Title/Abstract] AND "Eastern"[Title/Abstract]) OR "Maghreb"[Title/Abstract] OR "Maghrib"[Title/Abstract] OR "Arab*"[Title/Abstract] OR "Bedouin*"[Title/Abstract] OR "Gulf Cooperation Council"[Title/Abstract] OR "GCC"[Title/Abstract] OR "Middle East"[Title/Abstract] or “MENA”[Title/Abstract] OR "Middle East and North Africa"[Title/Abstract]) | |
| Web of Science: 1476 | 16th November 2025 |
| (elderly OR (older population) OR (older adults) OR geriatric)<br>AND<br>((accidental falls) OR falling OR falls OR (accidental slip) OR (unintentional fall) OR (loss of balance))<br>AND<br>(Qatar OR Bahrain OR Oman OR (Saudi Arabia) OR Kuwait OR (United Arab Emirates) OR Yemen OR Egypt OR Jordan OR Lebanon OR Syria OR Iraq OR Algeria OR Libya OR Morocco OR Tunisia OR Djibouti OR Sudan OR South Sudan OR Pakistan OR Palestine OR "middle east" OR "Arabs" OR UAE OR U.A.E OR Emirat* OR (Gulf Cooperation Council) OR GCC OR MENA OR (Middle East and North Africa) | |

## Box S2: Search Strategy—Supplementary search

| Search engine | Date |
|---|---|
| Google Scholar: 1539 | |
| Search 1a: | 16th November 2025 |
| With all of the words: ~Accidental falls<br>Exact phrase: Elderly<br>With at least one of the words: MENA "Middle East" "North Africa" Algeria Bahrain Djibouti Egypt Iraq Jordan Kuwait Lebanon Libya Morocco Oman | |
| Search 1b: | 16th November 2025 |
| With all of the words: ~Accidental falls<br>Exact phrase: Elderly<br>With at least one of the words: GCC “Gulf Cooperation Council” Maghreb Pakistan Palestine Qatar “Saudi Arabia” KSA Sudan Syria Tunisia “United Arab Emirates” UAE Yemen | |
| Search 2a: | 17th November 2025 |
| With all of the words: ~Fear of falling<br>Exact phrase: Elderly<br>With at least one of the words: MENA "Middle East" "North Africa" Algeria Bahrain Djibouti Egypt Iraq Jordan Kuwait Lebanon Libya Morocco Oman | |
| Search 2b: | 17th November 2025 |
| With all of the words: ~Fear of falling<br>Exact phrase: Elderly<br>With at least one of the words: GCC “Gulf Cooperation Council” Maghreb Pakistan Palestine Qatar “Saudi Arabia” KSA Sudan Syria Tunisia “United Arab Emirates” UAE Yemen | |
| Search 3a: | 19th November 2025 |
| With all of the words: ~ Elderly<br>Exact phrase: Risk of falls<br>With at least one of the words: MENA "Middle East" "North Africa" Algeria Bahrain Djibouti Egypt Iraq Jordan Kuwait Lebanon Libya Morocco Oman | |
| Search 3b: | 19th November 2025 |
| With all of the words: ~ Elderly<br>Exact phrase: Risk of falls<br>With at least one of the words: GCC “Gulf Cooperation Council” Maghreb Pakistan Palestine Qatar “Saudi Arabia” KSA Sudan Syria Tunisia “United Arab Emirates” UAE Yemen | |
| Search 4a: | 19th November 2025 |
| With all of the words: ~ Elderly, ~Accidental, ~Fear of falling<br>Exact phrase: Risk of falling, Risk of falls, Elderly<br>With at least one of the words: GCC “Gulf Cooperation Council” Maghreb Pakistan Palestine Qatar “Saudi Arabia” KSA Sudan Syria Tunisia “United Arab Emirates” UAE Yemen | |
| Search in Arabic | 8th December 2025 |
| "سقوط" AND "كبار السن" AND "سوريا"8<br>"سقوط" AND "كبار السن" AND "سودان"1<br>"سقوط" AND "كبار السن" AND "اليمن"6<br>"سقوط" AND "كبار السن " AND "ليبيا"3 | |
| Google: 70 | |
| Search in google | 8th December 2025 |

("older adults" OR "elderly") (falls OR "accidental falls") ("national survey" OR "government report" OR "ministry of health" OR "health statistics") filetype:pdf (site:moh.gov.bh OR site:moph.gov.lb OR site:moph.gov.qa OR site:moh.gov.om OR site:moh.gov.kw OR site:moph.gov.ps OR site:moh.gov.sa OR site:mohap.gov.ae OR site:health.gov.eg OR site:sana.dz OR site:health.gov.jo OR site:health.gov.ly OR site:sante.gov.ma OR site:santetunisie.rns.tn OR site:iraq-mohel.org OR site:moph.dj OR site:.dz OR site:.bh OR site:.dj OR site:.eg OR site:.ir OR site:.iq OR site:.jo OR site:.kw OR site:.lb OR site:.ly OR site:.ma OR site:.om OR site:.qa OR site:.sa OR site:.ps OR site:.sd OR site:.sy OR site:.tn OR site:.ae OR site:.ye) -site:ncbi.nlm.nih.gov -site:researchgate.net -site:pubmed.ncbi.nlm.nih.gov

Table S2: Characteristics of included studies

| Reference | Country | Response rate % | Study Design | Sampling | Year of data collection | Description | Mean Age (SD), Range | Study sample size | Sex n (%) | Outcomes | Assessment tool |
|---|---|---|---|---|---|---|---|---|---|---|---|
| **HIGH-INCOME COUNTRIES** | | | | | | | | | | | |
| Abdelhafez, 2023 (1) | Saudi Arabia | 86.95 | NR | Randomized controlled intervention | 2022 | Elderly patients with balance and gait issues selected from the National Institute of Neuromotor Systems. Intervention and control groups. | 64.74 | 60 | M: 29 (48.3)<br>F: 31(51.7) | Gait | TUGT |
| | | | | | | | | | | Balance | TIT |
| Alabdullgader, 2021 (2) | Saudi Arabia | 96.1 | CS | NR | 2019 | Elderly patients visiting 10 out of 19 randomly selected primary health centers in Unaizah City, Qassim Province. | 70.3 (8.18) for males 68.6 (6.8) for females, 60-90 | 269 | M: 133 (49.4)<br>F: 136 (50.6) | Multi-factorial fall risk | MAHC-10 |
| Alharbi, 2023 (3) | Saudi Arabia | NR | NR | Convenience | 2021** | Ambulatory community-dwelling individuals were recruited from two rehabilitation centers (general government and private sector hospitals). | ≥65 | 114 | M: 73 (64.1)<br>F: 41 (35.9) | Balance | BBS |
| | | | | | | | | | | Balance | 5STS |
| | | | | | | | | | | Fear of falling | 5-point likert scale |
| | | | | | | | | | | Gait | TUGT |

| | | | | | | | | | | | |
|---|---|---|---|---|---|---|---|---|---|---|---|
| Alshammari, 2018 (4) | Saudi Arabia | NR | CS | Convenience | 2015-2016 | Elderly patients who visited the largest mosques, malls, hospitals, and health centers in Riyadh, which was divided into five sections: North, South, Central, East, and West. | ≥60 | 357 | M: 168 (47.1)<br>F: 189 (52.9) | Multi-factorial fall risk | MAHC-10 |
| | | | | | | | | | | Multi-factorial fall risk | MFS |
| Alshehri, 2024(5) | Saudi Arabia | NR | National CS | NR | 2022 | Elderly individuals, both infected and non-infected with COVID-19, from the central, western, and eastern provinces of Saudi Arabia were included in the study. Subjects were excluded if they had neurological disorders, peripheral neuropathy, vestibular impairments, or had undergone arthroplasty. Additionally, those taking medications that could affect balance were also excluded. | 60-75 | 86 | NR | Multi-factorial fall risk | MAHC-10 |
| Altaymani, 2023(6) | Saudi Arabia | NR | CS | Convenience | 2021** | Elderly patients admitted to the acute care unit at King Salman Armed Forces Hospital (KSAFH). | 60-89 | 150 | M: 65 (43.3)<br>F: 85 (56.7) | Multi-factorial fall risk | MFS |

| | | | | | | | | | | | |
|---|---|---|---|---|---|---|---|---|---|---|---|
| Abd El-Kafy, 2024(7) | Saudi Arabia | NA | RCT | Convenience | 2022 | Elderly patients included in the experimental and control groups of an RCT conducted by the Department of Medical Rehabilitation Sciences at Umm Al-Qura University, Makkah, and Badghish Rehabilitation & Health Care Center, Jeddah, were included if they were medically stable and scored more than 13.5 on the Timed Up and Go test. Participants were excluded if they had any neurological conditions, walking difficulties, balance deficits, muscle paralysis, lower limb joint stiffness or bony deformities, seizures, lower limb joint arthritis, visual or auditory impairments, required assistive walking devices, vertigo, or vestibular problems. | 66.53 (3.82), 60-70 | 57 | M: 23 (79.3) F: 6 (20.7) | Fall risk | "Risk of Fall test" |
| | | | | | | | | | | Gait | TUGT |
| Alzaabi, 2022 (8) | UAE | NR | CS | Convenience | 2020-2021 | Community-dwelling elderly were recruited from community centers. Individuals with a history of | 75 (3.6) | 100 | M: 50 (50) F: 50 (50) | Fear of falling | VAS |

| | | | | | | | | | | |
|---|---|---|---|---|---|---|---|---|---|---|
| | | | | | | smoking, chronic obstructive pulmonary disease, chronic cardiac disease, asthma, lower limb surgery (e.g., hip or knee arthroplasty), lower limb deformity requiring a heel lift on their shoe, and visual or vestibular problems were excluded. | | | | Gait | TUGT |
| Abd Elkader, 2022 (9) | Saudi Arabia | NR | CS | Purposive | 2018-2019 | Elderly aged 60-69 years who were community-ambulant, overweight or obese and had symptoms of musculoskeletal pain in the lower limb or spine or both. Individuals with recent injury, having actute pain or with significant limitation to mobility were excluded. | 63.1 (2.5); 60-69 | 150 | M: 108 (72) F: 42 (28) | Gait | TUGT |
| Abdelaal, 2022 (10) | Saudi Arabia | NR | Single-Blinded Randomized control Trial | NR | 2019 | Elderly with type II diabetes and diabetic peripheral neuropathy were included. Individuals with type 1 diabetes, younger than 60 or older than 80 years old, following diet regimen, with malnutrition (below 11 points on the MNA scale), recently involved in an exercise training program within the last 6 months, underwent surgical | ≥60 | 62 | 62 (100) | Balance & stability | POMA |

| | | | | | | | | | | | |
|---|---|---|---|---|---|---|---|---|---|---|---|
| | | | | | | intervention within the last 6 months, with feet ulcers, individuals with serious cardiovascular insult or severe complications that can impact their safety, performance and affects study outcomes were excluded. | | | | | |
| Al Harbi, 2025 (11) | Saudi Arabia | NR | CS | NR | 2018-2019 | Elderly with an ability to communicate effectively, walk independently or with assistive devices at the 3 tertiary hospitals: King Abdilaziz Hospital, King Fahad Hospital and King Abdullah Medical Center and the Geriatric Society Center in Jeddah. | 66.5 (6.9) | 170 | M: 77 (45.3) F: 93 (54.7) | Gait | TUGT |
| | | | | | | | | | | Fear of falling | FES-I |
| Alahmari, 2024 (12) | Saudi Arabia | NR | CS | NR | 2022-2023 | Elderly individuals both with and without hip osteoarthritis who were able to understand and follow study instructions were recruited from outpatient orthopedic clinics in Abha, Saudi Arabia. Individuals were excluded if they had other musculoskeletal disorders that could affect balance and mobility, such as severe knee osteoarthritis or lower limb | ≥65 | 86 | NR | Gait | TUGT |

| | | | | | | | | | | | |
|---|---|---|---|---|---|---|---|---|---|---|---|
| | | | | | | fractures. Neurological conditions which could impair balance, were also grounds for exclusion. | | | | | |
| Al-Amoud, 2023 (13) | Saudi Arabia | NR | CS | All target | 2012-2020 | Elderly attending the older people health clinics at Primary Health Care Clinics in Saudi Arabia. | ≥60 | 193715 | M: 93886 (48.5)<br>F: 99829 (51.5) | Gait | Gait assessment Up and go |
| | | | | | | | | | | Balance | Romberg Test |
| Alamri, 2023 (14) | Saudi Arabia | NR | CS | NR | 2021 | Community-dwelling older adults in Jeddah | 60-105 | 403 | M: 132 (32.8)<br>F: 271 (67.2) | Fear of falling | Single-item questionnaire |
| Alanazi, 2023 (15) | Saudi Arabia | NR | CS | Random | 202 3 | Elderly aged 60 or more who visited one of 10 primary healthcare centers in Tabuk city, Saudi Arabia were included. | ≥60; 60-69 | 296 | M: 98 (33.1)<br>F: 198 (66.9) | Multi-factorial fall risk | MAHC-10-Arabic version |
| Alasmari, 2023 (16) | Saudi Arabia | NR | CS | Convenienc e | 2022 | Elderly diagnosed with type II diabetes attending the Primary healthcare centers of the Ministry of National Guard-Health affairs in Jeddah. | >60 | 309 | M: 165 (53.2)<br>F: 145 (46.8) | Fear of falling | FES |
| Alharbi, 2023 (17) | Saudi Arabia | NR | Validation | Convenienc e | 2022 | ndependent elderly aged ≥65, living in the community, who could speak Arabic and follow the examiner's instructions visiting the two outpatient physical therapy departments in Saudi Arabia. Individuals | ≥65 | 110 | M: 72 (65.5)<br>F: 38 (34.5) | Risk | Self-rated FRQ |
| | | | | | | | | | | Balance | BBS |
| | | | | | | | | | | | 5STS |

| | | | | | | | | | | | |
|---|---|---|---|---|---|---|---|---|---|---|---|
| | | | | | | with neurological or orthopedic disorders that disturbed their walking and balance, uncontrolled hypertension, and cardiovascular disease were excluded. | | | | Gait | TUGT |
| Alharbi, 2025a (18) | Saudi Arabia | NR | CS | Convenience | 2023-2024 | Elderly attending community centers and healthcare providers, able to read and understand Arabic and capable of walking independently in five regions of Saudi Arabia. | ≥65 | 473 | M: 195 (41.2)<br>F: 278 (58.8) | Risk | FRQ-Arabic |
| | | | | | | | | | | Fear of falling | Single-item questionnaire |
| AlHarkan, 2023 (19) | Saudi Arabia | NR | CS | Systematic random | 2021-2022 | Elder adults recruited at the Family and Community Medicine Centre at Imam Abdulrahman bin Faisal University (IAU) and King Fahad Hospital of the University (KFHU) outpatient departments in Khobar city, Saudi Arabia. Elderly who were able to walk independently were included. Patients who had acute illness or were diagnosed with neurocognitive disorders, such as dementia, and Parkinson's disease, and/or those who cannot recall their medications, | >60 | 387 | M: 242 (62.53)<br>F: 145 (37.47) | Risk | STEADI |
| | | | | | | | | | | Fear of falling | Single-item questionnaire |

| | | | | | | | | | | | |
|---|---|---|---|---|---|---|---|---|---|---|---|
| Ali, 2024 (20) | Saudi Arabia | NR | CS | NR | 2023 | Elderly individuals in the Madinah region. | 67.91 (9.06) | 189 | M: 103 (54.5)<br>F: 86 (45.5) | Risk | Subjective question "Fall Risk" |
| Aljarbou, 2025 (21) | Saudi Arabia | NR | CS | Random | 2023** | Elderly nursing home residents at the Ministry of Human Resource and Social Development. | 74.1 (10.9, 60-110 | 323 | M: 236 (73.1)<br>F: 87 (26.9) | Risk | STEADI |
| Alkhamis, 2025 (22) | Saudi Arabia | 87.5 | CS | Convenience | 2023-2024 | Community-dwelling older adults attending the outpatient services at the Department of Geriatrics and Physical Medicine at King Khalid University, KSA | 72.54 (6.32) | 84 | M: 36 (42.9)<br>F:48 (57.1) | Balance | BBS |
| | | | | | | | | | | Fear of falling | FES-I |
| Almarwani, 2024 (23) | Saudi Arabia | NR | CS | Convenience | 2023 | Elderly community dwelling who were able to walk independently with or without an assistive device recruited from the King Salman Centre, Riyadh, Saudi Arabia. Exclusion criteria included the presence of uncompensated presence of hearing, visual and cognitive impairments. | 63.77 (4.74) | 104 | M: 49 (47.1)<br>F: 55 (52.9) | Gait | TUGT |
| | | | | | | | | | | Balance | BBS |
| Almarwani, 2024 (24) | Saudi Arabia | NR | CS | Convenience | 2022** | Community-dwelling Saudi older women who self-reported ability to independently walk a household distance with or without an assistive device, | 63.98 (4.9) | 150 | F: 150 (100) | Gait | TUGT |
| | | | | | | | | | | Balance | BBS |
| | | | | | | | | | | Fear of falling | FES-I |

| | | | | | | | | | | | |
|---|---|---|---|---|---|---|---|---|---|---|---|
| | | | | | | and without the assistance of another person. | | | | | |
| Almarwani, 2025 (25) | Saudi Arabia | NR | CS | NR | 2023 | Elderly attending the King Salman Social Center who were able to walk independently with or without the use of assistive device. | 69.54 (3.48) | 123 | M: 66 (53.7)<br>F: 57 (46.3) | Balance | 5STS<br>SLS |
| | | | | | | | | | | Fear of falling | FES-I Icon-Arabic version |
| Alqahtani, 2022 (26) | Saudi Arabia | NR | CS | NR | 2020** | Elderly community dwelling adults who were able to walk independently and able to follow instructions were recruited from the Riyadh and Madinah area. | 66.2 (6.2), 60-85 | 140 | M: 74 (52.8)<br>F: 64 (45.7) | Balance | BBS |
| | | | | | | | | | | Balance & Gait | Mini-BEST - Arabic version |
| | | | | | | | | | | Fear of falling | FES-I |
| AlQahtani, 2024 (27) | Saudi Arabia | NR | Clinical trial | Convenience | 2022** | Individuals experiencing balance impairment, selected from AlMareefa Medical College, who had encountered at least one instance of falling within the preceding year. Additionally, participants were required to exhibit a certain | ≥65 | 90 | M: 54 (60.0)<br>F: 36 (40.0) | Balance | BBS |
| Alshehri, 2025 (28) | Saudi Arabia | NR | CS | Purposive | 2023** | The experimental group were elderly individuals aged 65 years and older who were independent with adequate cognition diagnosed with Chronic nonspecific neck pain (CNSNP). Individuals with a history of cervical spine | ≥65 | 186 | NR | Gait | TUGT |
| | | | | | | | | | | Balance | BBS |

| | | | | | | | | | | | |
|---|---|---|---|---|---|---|---|---|---|---|---|
| | | | | | | surgery, neurological disorders, vestibular conditions, severe osteoarthritis or any other musculoskeletal conditions that could affect balance were excluded. The control group had healthy individuals aged 65 years and older, with no history of neck pain or musculoskeletal disorders in the previous six months. | | | | | |
| El-Bagalaty, 2023 (29) | Saudi Arabia | NR | CS | NR | 2021 | Community-dwelling older adults who were diagnosed with mild or moderate SARS-CoV-2 infection and discharged in the last 6 months were eligible. Controls were elderly with no previous signs or symptoms of SARS-C0V-2. Individuals were excluded from the study if they were unable to stand or walk independently for at least 10 m without assistive devices or needed help with activities of daily living; had a history of falling that caused musculoskeletal trauma; had received prior balance exercises or physical therapy; | ≥60 | 64 | NR | Balance | BBS |

| | | | | | | | | | | | |
|---|---|---|---|---|---|---|---|---|---|---|---|
| | | | | | | had a history of systemic or neurological diseases that might affect balance; or were unable to comprehend and follow verbal instructions. | | | | | |
| Hasan, 2024 (30) | Saudi Arabia | NR | Validation | Convenience | 2022** | Elderly community-dwelling participants who were, medically stable, and could ambulate independently (with or without assistive aid) from various communities and settings such as primary healthcare centers, social events, and community gatherings were included in the study | 66.60 (5.76) | 207 | M: 131 (63.2)<br>F: 76 (36.7) | Fear of falling | MFES-Arabic version |
| Ismail, 2024 (31) | UAE | NR | CS | NR | 2023 | All hemodialysis patients attending the hemodialysis unit at the medical center in Ajman, UAE. | >61 | 19 | NR | Fear of falling | JHFRAT |
| Moawd, 2022 (32) | Saudi Arabia | NR | RCT | Random | 2020 | Older adults recruited in the outpatient clinic in the College of Applied Medical Science, PSAU, Saudi Arabia. They moved independently, had no chronic medical problems and did not do moderate or | >65 | 49 | NR | Gait | TUGT |
| | | | | | | | | | | Balance | BBS |

| | | | | | | | | | | | |
|---|---|---|---|---|---|---|---|---|---|---|---|
| | | | | | | vigorous activity that were incompatible with low-to-moderate intensity exercise, had no cognitive impairments were included. In contrast, participants with severe motor and balance performance deficits, stroke, Parkinson's disease, vestibular diseases, and severe arthritis in the lower extremities were excluded from the study. | | | | Balance & Gait | TIT |
| | | | | | | | | | | Fear of falling | Single item questionnaire |
| Reddy, 2023 (33) | Saudi Arabia | NR | CS | Convenience | 2022** | Healthy individuals aged 40-80 were selected from university campus and the local community. Individuals with a history of musculoskeletal injury, diabetes, stroke, dizziness, vestibular dysfunction or surgical hardware were excluded. | 69.53 (4.32) | 100 | M: 59 (59)<br>F: 41 (41) | Gait | TUGT |
| Yasmeen, 2025 (34) | Saudi Arabia | NR | CS | NR | 2023-2024 | Community dwelling elderly in Jazan, southwestern province of Saudi Arabia. | 68.9 (6.0) | 391 | M: 226 (57.8)<br>F: 165 (42.2) | Risk | STEADI |
| **UPPER-MIDDLE INCOME COUNTRIES** | | | | | | | | | | | |
| Al-Ardhi, 2022 (35) | Iraq | NR | Correlational | Purposive | 2021-2022 | Elderly diabetic patients at the Endocrinology and Diabetes Center affiliated with Al-Hussein Teaching Hospital in Al-Muthanna Governorate. | 67.73 (7.001), 60-94 | 107 | M: 53 (49.5)<br>F: 54 (50.5) | Balance | BBS |

| | | | | | | | | | | | |
|---|---|---|---|---|---|---|---|---|---|---|---|
| Qader, 2013 (36) | Iraq | NR | Case control | Random | 2012 | Elderly "control" respondents, recruited from six Non-Governmental Organization (NGO) that provide social and medical services to the community in different areas of Baghdad. | 66.28 (NR), 60-85 | 358 | M: 166 (46.4)<br>F: 192 (53.6) | Fear of falling | NR |
| Muhaidat, 2022 (37) | Jordan | NR | CS | Convenience | 2020** | Community-dwelling elderly individuals were recruited from shopping malls, local community centers, personal contacts, and the waiting rooms of outpatient clinics at the University of Jordan Hospital in the capital, Amman. Participants were excluded if they had any health condition affecting their ability to walk 10 meters or if they were unable to provide consent. | 60-83 | 120 | M: 39 (32.5)<br>F: 81 (67.5) | Fear of falling | Single-item questionnaire |
| Alghwiri, 2024 (38) | Jordan | NR | Reliability | NR | 2023 | Community-dwelling individuals who were 60 years or older and with common chronic conditions such as diabetes and hypertension that did not affect their general health. | 67.2 (5.9) | 75 | M: 28 (37.3)<br>F: 47 (62.7) | Fear of falling | FES-I |
| **LOWER-MIDDLE INCOME COUNTRIES** | | | | | | | | | | | |

| | | | | | | | | | | | |
|---|---|---|---|---|---|---|---|---|---|---|---|
| Abdelkhalik, 2023(39) | Egypt | NR | CS | Purposive | 2021-2022 | Elderly individuals in the Ear, Nose & Throat (ENT) and internal medicine outpatient clinics at Elsalam hospital, Port Said City. Elderly who were at risk of fall, had a chronic disease and able to walk 10 meters were included. Elderly with mental disorders, postural hypotension and who had previous hip replacement surgery or a history of lower extremities fracture in the last 12 months were excluded from the research. | 68.5 (6.7) | 77 | M: 49 (63.6) F: 28 (36.4) | Balance | BBS |
| | | | | | | | | | | Multi-factorial fall risk | HFRM |
| Adly, 2020 (40) | Egypt | NR | CS | NR | 2018 | Patients admitted to a geriatric inpatient department in Ain Shams University Hospital, Cairo. | 68.67 (7.33) | 190 | M: 94 (49.5) F: 96 (50.5) | Multi-factorial fall risk | HFRM |
| | | | | | | | | | | Multi-factorial fall risk | JH-FRAT |
| | | | | | | | | | | Multi-factorial fall risk | Schmid-FRAT |
| | | | | | | | | | | Multi-factorial fall risk | MFS |

| | | | | | | | | | | | |
|---|---|---|---|---|---|---|---|---|---|---|---|
| Al Tehewy, 2015 (41) | Egypt | NA | Observational longitudinal | Random | 2009 | Elderly patients admitted to the internal medicine department at Ain Shams University Hospital. | 67.6 (6.7), 60-90 | 411 | M: 224 (54.5)<br>F: 187 (45.5) | Multi-factorial fall risk | MFS |
| Ali Ibrahim, 2023(42) | Egypt | NR | CS | Purposive on all target population | 2023 | Elderly community dwellers diagnosed with cataract at outpatient clinics of the General Ophthalmology Hospital, previously Farouk Hospital, in Alexandria. They had no history of ophthalmic surgeries or glaucoma, no active state of orthopedics, and no neurological disorders such as cerebral vascular stroke and/or Parkinson's disease. Note: All ophthalmologic problems are treated at the facility. | 71.82 (7.68), 60-87 | 100 | M: 47 (47)<br>F: 53 (53) | Balance | BBS |
| | | | | | | | | | | Fear of falling | SFES-I |
| Amer, 2018 (43) | Egypt | NR | CS | Convenience | 2016** | Elderly patients in Nursing homes in Ain Shams, Cairo AND Elderly dwelling in community in Cairo. | ≥60 | 200 | M/F (Nb NR) | Fear of falling | Single-item questionnaire |
| | | | | | | | | | | Gait | TUGT |
| El-Gilany, 2011 (44) | Egypt | 82 | CS | All target population | 2010 | Elderly living in Menut Sandob village, Mansoura region. | ≥60 | 766 | M/F (Nb NR) | Multi-factorial fall risk | FROP-COM |

| | | | | | | | | | | | |
|---|---|---|---|---|---|---|---|---|---|---|---|
| El-Gilany, 2013 (45) | Egypt | NR | Pre-Post Intervention | Convenience | 2010-2011 | Elderly with history of fall within the last year living in Menyet Sandoub village, Mansoura region. | 69.6 (6.2), 60-91 | 63 | M: 21 (33.3)<br>F: 42 (66.7) | Balance | BBS |
| El-Kawaly, 2016 (46) | Egypt | NR | CS | Convenience | 2014-2015 | Elderly living at four different nursing homes in Cairo. | 72.15 (8.78) | 100 | M: 49 (33.3)<br>F: 51 (66.7) | Gait | TUGT |
| El-Rahman, 2014 (47) | Egypt | NR | CS | NR | 2013-2014 | Elderly living in Dar Al Mogama governmental elderly home in Damanhur, El-Behaira Governorate- Egypt. It is the only elderly home in the governorate. | 68.8 (7.1), 60-84 | 114 | M: 60 (52.6)<br>F: 54 (47.4) | Balance | BBS |
| | | | | | | | | | | Fear of falling | VAS |
| | | | | | | | | | | Gait | TUGT |
| Elsamahy, 2019 (48) | Egypt | NR | CS | Convenience | 2017-2018 | Elderly living in Grise village, which is affiliated to Ashmoon city in El- Monofya Governorate. Free from dementia. | ≥60 | 272 | M: 133 (48.9)<br>F: 139 (51.1) | Fear of falling | NR |
| Hassan, 2023a(49) | Egypt | NR | CS | NR | 2021** | Community-dwellers elderly from geriatric homes in urban and rural areas in Nile Delta Region (Damietta, Al Dakahleya, Kafr Al Shaikh, Al Monofeya, Al Gharbeya Governorates), Egypt. Participants were excluded if they had mobility or intellectual impairment. | 68.19 (7.26) | 183 | M: 92 (41.3)<br>F: 91 (40.8) | Fear of falling | FES-I |

| | | | | | | | | | | | |
|---|---|---|---|---|---|---|---|---|---|---|---|
| Ibrahim, 2019 (50) | Egypt | NR | CS | Convenience | 2017-2018 | Elderly participating in geriatric clubs benefit from social and healthcare services offered in Assiut city. | 73.18 (14.52), 60-89 | 88 | M: 36 (40.9) F: 52 (59.1) | Balance | BBS |
| Ismail, 2018 (51) | Egypt | NR | Quasi-experimental | Convenience | 2016-2017 | Elderly residents from 7 geriatric homes in different districts in Cairo. | 69.3 (6.2) | 120 | M: 59 (49.2) F: 61 (50.8) | Multi-factorial fall risk | FRAS |
| | | | | | | | | | | Fear of falling | Subjective questionnaire "Worried about falling" |
| Khater, 2012 (52) | Egypt | 100 | Prospective | Convenience | 2009-2010 | Elderly residents in nursing homes who exhibit both mobility and cognitive competence. | 71.9 (7.2), 60-90 | 84 | M: 36 (42.9) F: 48 (57.1) | Gait | TUGT |
| Mahmoued, 2014 (53) | Egypt | NR | CS | Purposive | 2012-2013 | Elderly residing at home in the rural area of Zagazig district, who are not bedridden or mentally impaired, and maintain independence in performing their daily activities. | 60-80 | 236 | M: 106 (44.9) F: 130 (55.1) | Fear of falling | MFES |
| | | | | | | | | | | Gait | TUGT |
| Makhlouf, 2000 (54) | Egypt | 13 to 69 | CS | Convenience | 1998-1999 | Elderly individuals from six out of fourteen geriatric institutions in Alexandria were included in the study, with exclusion criteria applied to those with advanced cognitive impairment. | 73.5 (7.46) for M 73.4 (7.69) for F | 165 | M: 62 (37.6) F: 103 (62.4) | Balance & Gait | TIT |

| | | | | | | | | | | | |
|---|---|---|---|---|---|---|---|---|---|---|---|
| Mohamed, 2019 (55) | Egypt | NR | Quasi-experimental | Purposive | 2017-2018 | Elderly from three geriatric homes in minia city (Dar Omar Bn El khatab, Dar El Qedesa Hena, and Dar El raee el saleh) who are able to exercise and carry out daily activities. | 67.3 (6.4), 60-85 | 80 | M: 49 (61.25) F: 31 (38.75) | Balance | BBS |
| Mohammed, 2021 (56) | Egypt | NR | CS | Convenience | 2019-2020 | Elderly residents living in one of three geriatric homes and at the outpatient clinics of Minia City-Minia Government, attending the diabetic, orthopedic, ophthalmic, and medical clinics. | 66.1 (5.6) | 384 | M: 186 (48.4) F: 198 (51.6) | Gait | TUGT |
| Mohsen, 2019 (57) | Egypt | NR | Quasi-experimental | Random | 2017-2018 | Elderly individuals with a history of falls residing in two villages of Ghemreen village and Manshat Sultan village in Menoufia governorate. | ≥60 | 130 | M/F (Nb NR) | Fear of falling | MFES |
| Naeem, 2020 (58) | Egypt | NR | CS | Convenience | 2018-2019 | Elderly patients from five primary healthcare centers on 6th October City, located in Greater Cairo Governorate: Al Hosary, Al Shabab, Al Mostakbal, Al Reaya, and Haram City Primary Healthcare Centers. | 66.84 (5.86), 60-90 | 230 | M: 102 (44.3) F: 128 (55.7) | Gait | TUGT |
| Rasheedy, 2021 (59) | Egypt | NA | CS | NR | 2016-2018 | Older adults admitted to the geriatric department of Ain | 69.45 (7.80) | 206 | M: 98 (47.6) F: 108 (52.4) | Gait | TUGT |

| | | | | | | | | | | | |
|---|---|---|---|---|---|---|---|---|---|---|---|
| | | | | | | Shams University Hospital in Cairo. | | | | | |
| Saleh, 2018 (60) | Egypt | NR | CS | Random | 2017-2018 | Elderly individuals residing in both urban and rural areas affiliated with Mansoura city. In urban areas, participants were recruited from four health centers: El-Hawar Health Center, Sandowb Center, Toriel Center, and Gedela Center. In rural areas, participants were drawn from four villages: Badaway Village, Meet Badr Khamis Village, Meet Ali Village, and Nekita Village. Eligible participants in both urban and rural settings were those who were not bedridden or reliant on a wheelchair for mobility. | 70.31 (7.05), 60-85 | 528 | M: 236 (44.7) F: 292 (55.3) | Balance | BBS |
| | | | | | | | | | | Fear of falling | MFES |
| Sharaf, 2008 (61) | Egypt | 100 | CS | All target population | 2006** | Older adults recruited from all government assisted living facilities (elderly homes) in Alexandria and 2 Arabic | 73.21 (8.86), 60-102 | 208 | M: 83 (39.9) F: 125 (60.1) | Fear of falling | MFES |

| | | | | | | | | | | | |
|---|---|---|---|---|---|---|---|---|---|---|---|
| | | | | | | speaking private facilities. They are independent or semi-independent and able to care for themselves, mobile with or without assistance, and free from any cognitive or physical impairment, psychiatric disease, or infection. | | | | Fear of falling | Single-item questionnaire |
| Salman, 2024(62) | Egypt | NR | CS | Convenience | 2022 | Independent elderly patients attending the outpatient internal medicine, orthopedics, dental, ophthalmology, ear, nose and throat and Rheumatology clinics at Suez Canal University hospitals. | ≥65 | 310 | M: 181 (58.4)<br>F: 129 (41.6) | Balance | BBS |
| | | | | | | | | | | Fear of falling | MFES |
| | | | | | | | | | | Gait | TUGT |
| Tawfik, 2021(63) | Egypt | 100 | Prospective | All target population | 2018-2019 | Inpatients from wards of Ain Shams University Hospitals, who underwent elective cardiac surgery and had no postoperative complication. | ≥60 | 180 | M: 137 (76.1)<br>F: 43 (23.9) | Gait | TUGT |
| Mahdi, 2017 (64) | Morocco | NR | NR | NR | 2014 | Patients diagnosed with rheumatoid arthritis at the department of rheumatology, at El Ayachi Hospital, Rabat. | ≥61 | 40 | NR* | Balance & Gait | TIT |
| | | | | | | | | | | Gait | TUGT |

| | | | | | | | | | | | |
|---|---|---|---|---|---|---|---|---|---|---|---|
| Adnan, 2022 (65) | Pakistan | NR | Single-Blinded Randomized control Trial | Convenience | 2021-2022 | Elderly population from primary care centers who were pre-frail according to fried's frailty phenotype and at a fall risk with a PPA composite score of 0 or above. They were not receiving palliative care, prescribed psychotropic, antiarrhythmic, or hypnotic medications, or diagnosed with heart disease, cancer, or severe depression.<br>The population included an intervention and a control group at baseline. Data was collected from the Management records of geriatric population of primary care centers of Rawalpindi and Islamabad. | 65.14 (3.65) | 54 | M/F (Nb NR) | Fall risk | NR |
| | | | | | | | | | | Fear of falling | NR |
| Anwar, 2023 (66) | Pakistan | NR | CS | Convenience | 2021** | Elderly females from the community in the Punjab province.<br>Elderly females with a history of repetitive falls, transient ischemic attacks and strokes, neurological and cognitive disorders, use of wheelchairs, | 60-80 | 350 | F: 350 (100) | Balance | BBS |
| | | | | | | | | | | Gait | TUGT |

| | | | | | | | | | | |
|---|---|---|---|---|---|---|---|---|---|---|
| | | | | | | and central nervous system neoplasms were excluded. | | | | |
| Bibi, 2023 (67) | Pakistan | NR | Interventional study | Convenience | 2022 | Elderly from two residential areas of Peshawar district, Khyber-Pakhtunkhwa, who have a sedentary lifestyle that is <2 hours activity in 24 hours, with normal to moderate cognitive functioning, able to follow instructions, and able to walk without taking assistance from another person.<br>Baseline data for the random control trail study. | 68.1 (50), 60-82 | 140 | M: 64 (46)<br>F: 76 (54) | Gait | TUGT |
| Gul, 2021 (68) | Pakistan | NR | CS | Convenience | 2014 | Elderly able to walk independently living in household from the Police Colony of Peshawar, Khyber Pakhtunkhwa. | ≥60 | 111 | M: 59 (53)<br>F: 52 (46.8) | Fall risk | Subjective questionnaire “Fall Risk Assessment” |

| | | | | | | | | | | | |
|---|---|---|---|---|---|---|---|---|---|---|---|
| Mehmood, 2016 (69) | Pakistan | NR | CS | Convenience | 2015 | Community-dwelling elderly individuals living independently in Faisalabad, who can walk without an assistive device (e.g., cane, crutch, walker, wheelchair) and without the help of another person. They did not have any common neurological or musculoskeletal disorders, or pain that could restrict movement. Elderly participants were recruited from Madinah Teaching Hospital and Allied Hospital.<br>Note: confusing statement: "People were excluded who had previously only one fall within past six months, for the sake of expanding the opportunity of choosing a specimen of elder people having repeated falls, deficit in cognition in terms of aphasia, severe visual and audible deterioration that might inhibit the member from performing the physical tests or answering of questionnaire". | ≥65 | 77 | M: 77 (100) | Multi-factorial fall risk | EFST |
| | | | | | | | | | | Gait | TUGT |

| | | | | | | | | | | | |
|---|---|---|---|---|---|---|---|---|---|---|---|
| Naseer, 2022 (70) | Pakistan | NR | CS | Convenience | 2020** | Elderly with from local Pakistani regions in Islamabad and Rawalpindi. Note: confusing definition of the population "Individuals with neurological deficiencies or comorbid conditions, a history of falls, fractures or any surgery were excluded from the study". However, all had injuries due to fall. | 69.76 (8.5), 60-108 | 184 | M: 103 (56) F: 81 (44) | Balance | BBS |
| Nisar, 2024 (71) | Pakistan | NR | CS | Convenience | 2022 | Elderly frail and non-frail patients in the outpatient department of physical therapy, as well as admitted to the medical ward at Ariz Memorial Hospital and DHQ Hospital, Kasur. Patients with the history of limb amputation, cognitive impairment, patients on wheelchair and in ICU were excluded. | ≥65 | 90 | M: 46 (51) F: 44 (49) | Balance | BBS |
| Shahid, 2020 (72) | Pakistan | NA | "Correlational" | Purposive | 2018 | Elderly patients from Railway General Hospital, Rawalpindi Eye Donors Organization (REDO) Eye Hospital, Water and Power Development | 70.03 (4.52) | 336 | M: 270 (80.35) F: 66 (19.65) | Multi-factorial fall risk | HFRM |

| | | | | | | | | | | | |
|---|---|---|---|---|---|---|---|---|---|---|---|
| | | | | | | Authority General Hospital, as well as Baghban and Mukhtar Ghulam Qadir (MGQ) old people's homes in Islamabad and Rawalpindi, who were able to walk 10 meters without assistance and understand instructions. | | | | Fear of falling | FES-I |
| Tariq, 2018 (73) | Pakistan | NR | CS | Convenienc e | 2016** | Geriatric population that suffered from musculoskeletal pain in different body part from either Allied Hospital or Madinah Teaching Hospital, Faisalabad. | 71 (7) | 71 | M: 31 (43.7)<br>F: 40 (56.3) | Balance | BBS |
| Zulfiqar, 2019 (74) | Pakistan | NR | CS | Convenienc e | 2017-2018 | Older adults who did not have any impairment recruited at Children Hospital, Lahore General Hospital and Nawaz Sharif Social Security Hospital in Lahore | 65-92 | 84 | M/F (Nb NR) | Fear of falling | MFES |
| Badrasawi, 2022 (75) | Palestine | 100 | CS | Convenienc e | 2020-2021 | Elderly individuals from the Hebron district who did not have any acute illnesses or severe hearing problems on the days of data collection. | ≥65 | 200 | M: 63(31.5)<br>F: 137 (68.5) | Gait | TUGT |
| Halaweh, 2016 a (76) | Palestine | NR | CS | Convenienc e | 2013-2014 | Community-dwelling elderly who were independent in indoor ambulation with or | 68.2 (6.74), 60-91 | 176 | M: 61 (34.7)<br>F: 115 (65.3) | Gait | TUGT |

| | | | | | | | | | | |
|---|---|---|---|---|---|---|---|---|---|---|
| | | | | | | without walking aids and living in the West Bank. | | | | | |
| Abbas, 2023 (77) | Lebanon | NR | Single-Blinded Randomized control Trial | Random | 2020 | Elderly, literate residents at Dar AlAjaza Allslamiah Hospital "DAIH" , diagnosed with moderate to severe dementia according to the MMSE (score <20) and stable at least 6 weeks prior to recruitment, with stable medication (dementia, mood stabilizer at least 3 months ago), acceptable hearing or visual acuity, and at least able to rise from a chair and walk for 6 meters with or without assistance. Elderly with any major neurological disorders (Parkinson, stroke) or any disease that might cause cognitive decline, any cardiovascular or systemic disease that affects the participants' ability to complete physical activity; substance abuse, and any involvement in therapeutic training activity at least 6 months prior recruitment were excluded. | 79 (6.67) | 20 | M: 10 (50)<br>F: 10 (50) | Gait | TUGT |
| | | | | | | | | | | Balance | BBS |

| | | | | | | | | | | | |
|---|---|---|---|---|---|---|---|---|---|---|---|
| Abd Elrahman, 2024 (78) | Egypt | NR | Descriptive correlational | Purposive | 2022** | Elderly aged 60 years or above, diagnosed with age related macular degeneration and able to communicate. | 67.26 (7.27) | 77 | M: 54 (70.1) F: 23 (28.6) | Fear of falling | MFES |
| Abo Elyazed, 2025 (79) | Egypt | NR | Randomised clinical study | Random | 2023** | Elderly with risk of falls at the Palestinian Red Crescent center in Cairo, Egypt. | ≥60 | 60 | NR | Balance | BBS |
| | | | | | | | | | | | Functional Reach Test |
| | | | | | | | | | | | Balance Y-MED |
| | | | | | | | | | | Gait | TUGT |
| Abdullah, 2025 (80) | Pakistan | NR | CS | Convenience | 2024 | Community dwelling elderly capable of following verbal instructions and walking independently without assistance from the Khyber Pakhtunkhwa Province, Pakistan. | 65.90 (3.84) | 176 | M: 116 (65.91) F: 60 (34.09) | Gait | TUGT |
| | | | | | | | | | | Balance | 5STS |
| | | | | | | | | | | Fear of falling | FES-I |
| Altaf, 2025 (81) | Pakistan | NR | CS | NR | 2023 | Elderly community dwelling individuals from both rural and urban areas across Pakistan. | 65.75 (5.99) | 387 | M: 226 (58.4) F: 161 (41.6) | Fear of falling | FES |
| Ammar, 2024 (82) | Pakistan | NR | RCT | Convenience | 2022** | Elderly of 65 years or older with a mini-mental state examination (MMSE) score of 26 or above and a minimum of one self-reported fall in the previous two years, or a BBS score between >41 and <52. | ≥65 | 64 | NR | Balance | BBS |

| | | | | | | | | | | | |
|---|---|---|---|---|---|---|---|---|---|---|
| | | | | | | The study excluded participants with vertigo, neurological disorders, eye diseases, significant walking or balance impairments, and physical limitations that might hinder hand mobility, and those using psychotropic medicines | | | | | |
| Arshad, 2022 (83) | Pakistan | NR | Validatio n | Purposive | 2019 | Elderly with fall history and absence of any pathological condition at Akbar Niazi Teaching Hospital (ANTH) were included. Patients with stroke and Parkinson disease, auditory and visual impairments and any recent head injury were excluded from the study. | 74.38 (9.46) | 16 | M: 7 (43.75)<br>F: 9 (56.25) | Balance | BBS |
| | | | | | | | | | | Fear of falling | FES-I |
| Badrasawi, 2021 (84) | Palestine | NR | CS | Random | 2019 | Elderly aged 60 years or more residing in the Se'ar and Bani Na'em, Hebron governance, in the south of the West Bank, Palestine. Individuals with acute illness, fractures of extremities or with a medical condition such as cachexia, severe edema or ascites and dementia were excluded. | 69.5 (5.7), 60-90 | 145 | M: 70 (48.3)<br>F: 75 (51.7) | Gait | TUGT |

| | | | | | | | | | | | |
|---|---|---|---|---|---|---|---|---|---|---|---|
| Battesha, 2022 (85) | Egypt | NR | RCT | NR | 2019 | patients with middle cerebral artery stroke confirmed by brain computed tomography scan with normal levels of blood glucose level and blood pressure levels. | 45-65 | 30 | NR | Balance | BBS |
| Dwedar, 2025 (86) | Egypt | NR | RCT | NR | 2024 | Elderly with Type II diabetes and a risk of fall living in elderly residences. | ≥65 | 42 | NR | Balance | BBS |
| | | | | | | | | | | Gait | TUGT |
| Edarous, 2025 (87) | Egypt | 84 | CS | NR | 2023** | Elderly living in geriatric homes. | 73.18 (7.09), 60-96 | 283 | M: 134 (47.3) F: 149 (52.7) | Multi-factorial fall risk | MFS |
| El Miedany, 2023 (88) | Egypt | NR | CS | All target | 2022-2023 | Egyptian postmenopausal women and men above 40 years of age, presenting with hip fragility fractures were included; however, those with pathological fractures or history of violent trauma were excluded from the study. Data were recovered from the Fracture Liaison Service National Register database | ≥60 | 326 | M: 108 (33) F: 218 (67) | Fall Risk | FRAS |
| El Miedany, 2023a (89) | Egypt | NR | CS | All target | 2022-2023 | All postmenopausal women and men above 50 years of age, presenting with one of the major osteoporosis fractures. However, those with | 70.1 (9.2) | 405 | M: 121 (30) F: 284 (70) | Fall Risk | FRAS |

| | | | | | | | | | | |
|---|---|---|---|---|---|---|---|---|---|---|
| | | | | | | pathological fractures or history of violent trauma were excluded from the study. Data were recovered from the Fracture Liaison Service National Register database. | | | | | |
| Mohammed Zein El-Abdeen Mohamed, 2025 (90) | Egypt | NR | Quasi-experimental intervention | Cluster | 2023** | Community dwelling frail elderly living in the Dakahlia governorate. | >60 | 70 | NR | Fear of falling | FES |
| Eldegwey, 2025 (91) | Egypt | NR | Single-Blinded Randomized control Trial | NR | 2024 | Elderly, clinically stable diabetic patients with peripheral neuropathy and a BMI of 25-32 kg/m2 who were able to communicate effectively were chosen from the outpatient clinics of Menouf hospital. | 65-75 | 60 | NR | Balance | BBS |
| Elgayar, 2025 (92) | Egypt | 70.8 | Prospective RCT | Random | 2024 | Elderly, clinically stable Type II diabetic men with glycated hemoglobin less than 7%, fair balance control; a BBS score between 21 and 40, and a BMI <30 kg/m2 recruited from the Department of Endocrinology at El Mahalla El Kobra General hospital in Egypt. | 65-75 | 76 | M: 76 (100) | Balance | BBS |
| | | | | | | | | | | Fall Risk | FRQ |

| | | | | | | | | | | | |
|---|---|---|---|---|---|---|---|---|---|---|---|
| Elsaid, 2024 (93) | Egypt | NR | CS | Convenience | 2022 | Elderly attending outpatient clinics in Menoufia University Hospital | 75 (9.7), 60-95 | 710 | M: 364 (51.3)<br>F: 346 (48.7) | Fear of falling | FES-I |
| Elshoura, 2025 (94) | Egypt | NR | Prospective interventional | Random | 2024 | Heart failure patients recruited from the cardiothoracic rehabilitation clinic at university hospitals. | 69 (4.5), 61–79 | 20 | M: 16 (80.0)<br>F: 4 (20.0) | Gait | TUGT |
| Fatima, 2022 (95) | Pakistan | NR | RCT | Convenience | 2020 | Elderly having a history of chronic dizziness with normal vestibular function, age related balance impairment with a BBS score between 35 and 45 and having a sufficient cognitive and communication skills. The participants with a history of impaired vestibular function, visual impairment, severe hearing loss, mental disorder that could restrain the ability to concentrate during therapy, other systematic diseases, and/or patients having any history of marked musculoskeletal impairment, lower limb injury, surgery other than age-related changes and balance disorder due to other neurological conditions like Parkinsonism, stroke, | >65 | 64 | NR | Balance | BBS |

| | | | | | | | | | | | |
|---|---|---|---|---|---|---|---|---|---|---|---|
| | | | | | | multiple sclerosis etc were excluded | | | | | |
| Ferhi, 2023 (96) | Tunisia | NR | CS | Convenience | 2022 | Older adults with sarcopenic obesity. Participants had to demonstrate a handgrip force less than 17 N, a gait speed under 1.0 m/s, be aged over 65 years, maintain the capacity for verbal communication with our team, and sustain physical independence. | ≥65 | 42 | NR | Gait | TUGT |
| | | | | | | | | | | Balance | Romberg tests |
| Ghous, 2024 (97) | Pakistan | NR | Single-Blinded Randomized control Trial | NR | 2022** | Elderly with standardized Mini-Mental state examination score >19 with a history of fall once or twice in the past year, identified being at medium risk of fall with a score of >36 on the Berg Balance scale, able to walk with or without assistive devices were included. However, participants with a progressive neurological disorder or vestibular disease like myasthenia gravis, stroke, multiple sclerosis or any severe inflammatory conditions, acute exacerbations, fractures and open wounds or patients declared unfit for physical | ≥65 | 28 | NR | Balance | BBS |

| | | | | | | | | | | |
|---|---|---|---|---|---|---|---|---|---|---|
| | | | | | | activities following medical assessment were excluded from the study. | | | | | |
| Hassan Ahmed , 2024 (98) | Egypt | NR | CS | Convenience | 2022-2023 | Elderly attending the outpatient clinics at Ain Shams University Hospital | ≥60 | 250 | NR | Fear of falling | FES-I |
| Hussein, 2024 (99) | Egypt | NR | CS | Convenience | 2020-2021 | Elderly diabetic from the inpatient wards of Ain Shams University hospitals. Bedridden, patients on medications that affect bone metabolism and/or medications for osteoporosis, bone diseases such as multiple myeloma, Paget's disease and recent fractures up to 6 months were excluded. | 66.8 (6.7) | 90 | M: 37 (41) F: 53 (59) | Gait | TUGT |
| | | | | | | | | | | Balance & Gait | TIT |
| Kandeel, 2024 (100) | Egypt | NR | CS | Random | 2022** | Elderly attending the vestibular clinics in El-Demerdash with a history of fall. | 70 (4.71) | 30 | M: 16 (53.33) F: 14 (46.67) | Fear of falling | FES-I |
| Kaynat, 2025 (101) | Pakistan | NR | CS | Convenience | 2023** | Elderly residing in three old age homes in Lahore, Pakistan. | 65-90 | 245 | M: 116 (47.3) F: 129 (52.7) | Fear of falling | FES-I |
| Khalil, 2024 (102) | Egypt | NR | Quasi-experimental intervention | Convenience | 2023 | Elderly with some level of mobility impairment necessitating the use of a device (cane or walker), be able to communicate, | ≥60 | 128 | NR | Fear of falling | FES-I |

| | | | | | | | | | | | |
|---|---|---|---|---|---|---|---|---|---|---|---|
| | | | | | | understand and follow simple instructions from four assisted living facilities in Alexandria, Egypt. Participants who exclusively used other walking aids were excluded from the study. Furthermore, individuals with visual problems that prevented adequate viewing of the character displayed on the mobile screen, any degree of paresis, a neurological disorder such as a stroke that prevented the performance of the proposed activity, a history of seizures, severe pain, or who were not mentally alert were also excluded | | | | Concern | FES-IAB |
| Khallaf, 2025 (103) | Egypt | NR | RCT | Random | 2023** | Community-dwelling individuals in the middle stage of Parkinson's disease were recruited from the outpatient clinic at the Faculty of Physical therapy, Cairo University, Egypt | 77.2 (4.6) | 40 | M: 34 (85)<br>F: 6 (15) | Gait | FGA |
| Magtouf, 2023 (104) | Tunisia | NR | Multicenter, Randomized, and | Convenience | 2022 | Individuals with obesity (BMI>30 kg/m2), a handgrip force (HF) lower than 17 N, a gait speed less than 1.0 m/s, and an age exceeding 65 years | ≥65 | 50 | NR | Gait | TUGT |
| | | | | | | | | | | Balance | Romberg tests |

| | | | | | | | | | | | |
|---|---|---|---|---|---|---|---|---|---|---|---|
| | | | Controlled Trial | | | | | | | | |
| Mousa, 2016 (105) | Egypt | NR | Case control | NR | 2012-2013 | Elderly who attended the Osteoporosis Detection Unit in Ain Shams University Hospital, Cairo, Egypt with poor mobility (TUG time >20 seconds) were included as cases and elderly individuals with good mobility (TUG time ≤ 20) were included as controls. | ≥60 | 138 | NR | Gait | TUGT |
| Nazeer, 2025 (106) | Pakistan | NR | CS | Convenience | 2024 | Elderly hypertensive patients attending the Bajwa hospital, Lahore with no neurological diseases, limb deformities or dizziness or had taken sedative or hypnotic drugs. | 71.7 (5.0) | 114 | F: 114 (100) | Balance | BBS |
| | | | | | | | | | | Gait | TUGT |
| | | | | | | | | | | Fear of falling | FES-I |
| Sayed, 2025 (107) | Egypt | NR | Prospective, observational | NR | 2023** | Elderly patients aged 60 years and above with Rheumatoid Arthritis from the Rheumatology Clinics of Alexandria Main University and from Internal Medicine department. | 68.4 (2.3), 65-73 | 60 | M: 26 (43.3)<br>F: 34 (56.7) | Fall Risk | Fall Risk Checklist |
| Waheed, 2024 (108) | Pakistan | NR | CS | Convenience | 2021 | Elderly with balance impairment and a history of | 71.5 (5.6) | 56 | M: 30 (53.57)<br>F: 26 (46.43) | Balance | BBS |

| | | | | | | | | | | |
|---|---|---|---|---|---|---|---|---|---|---|
| | | | | | | fall in the last one year and walking without aids for atleast 6 meters. | | | | Gait | TUGT |
| Zahra, 2023 (109) | Pakistan | NR | CS | Quota | 2021 | Healthy individuals aged 60 and above in various cities of Pakistan. | 70.36 (6.20) | 2004 | M: 1041 (51.95) F: 963 (48.05) | Balance | MRBT |

UAE-United Arab Emirates, NA-Not Applicable, NR-Not Reported, CS-Cross-sectional, RCT-Randomized controlled trial, M-Male, F-Female.

BBS-Berg Balance Scale, 5STS- Five times Sit-Stand, SLS-Single Leg stance, TIT-Tinetti assessment Tool, Mini BEST- Mini-Balance Evaluation Systems Test, MRBT- Modified Romberg Balance Test, POMA- Tinetti Performance Oriented Mobility Assessment scale, TUGT- Timed Up and Go test, MAHC-10- Missouri Alliance for Home Care fall risk assessment tool, JHFRAT- Johns Hopkins Fall Risk Assessment tool, FGA- Functional Gait Assessment, FRQ- Fall Risk Questionnaire, STEADI-Stopping Elderly Accidents, Deaths, and Injuries, MFS- Morse Fall Scale, FRAS- Falls Risk Assessment Score, FROP-Com-Fall Risk for Older People- Community Setting, HFRM -Hendrich II Fall Risk Model, EFST- Elderly fall screening test, Schmid Fall Risk Assessment (Schmid-FRAT), FES-Falls Efficacy Scale, FES-I- Fall Efficacy Scale-International, FES-IAB- Fall Efficacy Scale- International Avoidance Behavior, MFES -Modified Falls Efficacy Scale, SFES-I-Short Fall Efficacy Scale-international, Modified MFES-Arabic version- Falls Efficacy Scale-Arabic version,

In the “Mean Age (SD), Range” column, when studies reported a mean age but did not provide the standard deviation (SD), we indicated the SD as (NR). For studies that did not report mean age or SD, but only provided an age eligibility criterion (e.g., ≥60 or ≥65 years), we reported that threshold as given in the original publication. For studies that included participants younger than 60 years, only data reported separately for participants aged ≥60 years were extracted and included in the review.

*The sex distribution is reported for the whole population and not among the elderly population

** The year of data collection was not reported but was imputed by adjusting the year of publication based on the median difference between year of publication and the year of data collection (two years).

*** It includes health consequences and healthcare utilization

Table S3: Quality assessment of included studies

| Reference | Country | Conflict of Interest/Funding | 1 | 2 | 3 | 4 | 5 | 6 | 7a | 7b | 8 | 9 | 10 |
|---|---|---|---|---|---|---|---|---|---|---|---|---|---|
| HIGH-INCOME COUNTRIES | | | | | | | | | | | | | |
| Abdelhafez, 2023 (1) | Saudi Arabia | None | LR | LR | HR | LR | LR | HR | LR | NA | LR | LR | LR |
| Alabdullgader, 2021 (2) | Saudi Arabia | None | HR | LR | HR | LR | LR | LR | LR | NA | LR | LR | LR |
| Alharbi, 2023 (3) | Saudi Arabia | None | HR | LR | HR | HR | LR | HR | LR | LR | LR | LR | LR |
| Alshammari, 2018 (4) | Saudi Arabia | None | LR | LR | HR | HR | LR | HR | LR | NA | LR | LR | LR |
| Alshehri, 2024(5) | Saudi Arabia | None | LR | LR | HR | HR | LR | LR | LR | NA | LR | LR | LR |
| Altaymani, 2023(6) | Saudi Arabia | NR | HR | LR | HR | HR | LR | HR | LR | NA | LR | LR | LR |
| Abd El-Kafy, 2024(7) | Saudi Arabia | None [a] | HR | LR | HR | HR | LR | HR | LR | NA | LR | LR | LR |
| Alzaabi, 2022 (8) | UAE | None | LR | LR | HR | HR | LR | LR | LR | LR | LR | LR | LR |
| Abd Elkader, 2022 (9) | Saudi Arabia | None | HR | LR | HR | HR | LR | LR | LR | NA | LR | LR | LR |
| Abdelaal, 2022 (10) | Saudi Arabia | None [b] | HR | LR | LR | HR | LR | LR | LR | NA | LR | LR | LR |
| Al Harbi, 2025 (11) | Saudi Arabia | None | HR | LR | HR | HR | LR | LR | LR | HR | LR | LR | LR |
| Alahmari, 2024 (12) | Saudi Arabia | None | HR | HR | HR | HR | LR | LR | LR | NA | LR | LR | LR |
| Al-Amoud, 2023 (13) | Saudi Arabia | None [c] | LR | LR | HR | HR | LR | LR | NA | NA | LR | LR | LR |
| Alamri, 2023 (14) | Saudi Arabia | None | HR | LR | HR | LR | LR | LR | NA | HR | LR | LR | LR |
| Alanazi, 2023 (15) | Saudi Arabia | None | HR | LR | LR | HR | LR | HR | LR | NA | LR | LR | LR |
| Alharbi, 2023 (17) | Saudi Arabia | None | HR | LR | HR | HR | LR | LR | LR | NA | LR | LR | LR |
| Alharbi, 2025a (18) | Saudi Arabia | None | LR | LR | HR | HR | LR | LR | LR | HR | LR | LR | LR |
| AlHarkan, 2023 (19) | Saudi Arabia | None | HR | LR | LR | HR | LR | LR | LR | HR | LR | LR | LR |
| Ali, 2024 (20) | Saudi Arabia | None | HR | LR | HR | HR | LR | LR | LR | NA | LR | LR | LR |

| | | | | | | | | | | | | | |
|---|---|---|---|---|---|---|---|---|---|---|---|---|---|
| Aljarbou, 2025 (21) | Saudi Arabia | None | LR | LR | LR | HR | LR | LR | LR | NA | LR | LR | LR |
| Alkhamis, 2025 (22) | Saudi Arabia | None [d] | LR | LR | HR | LR | LR | LR | LR | LR | LR | LR | LR |
| Almarwani, 2024 (23) | Saudi Arabia | None | HR | LR | HR | HR | LR | LR | LR | NA | LR | LR | LR |
| Almarwani, 2025 (25) | Saudi Arabia | None | HR | LR | HR | HR | LR | LR | LR | LR | LR | LR | LR |
| Almarwani, 2024 (24) | Saudi Arabia | None | HR | LR | HR | HR | LR | LR | LR | LR | LR | LR | LR |
| Alqahtani, 2022 (26) | Saudi Arabia | None | HR | LR | HR | HR | LR | LR | LR | LR | LR | LR | LR |
| AlQahtani, 2024 (27) | Saudi Arabia | None | HR | LR | LR | LR | LR | LR | LR | NA | LR | LR | LR |
| Alshehri, 2025 (28) | Saudi Arabia | None [e] | HR | LR | HR | HR | LR | LR | LR | NA | LR | LR | LR |
| El-Bagalaty, 2023 (29) | Saudi Arabia | None | HR | LR | HR | HR | LR | LR | LR | NA | LR | LR | LR |
| Hasan, 2024 (30) | Saudi Arabia | None | HR | LR | HR | LR | LR | HR | NA | LR | LR | LR | LR |
| Ismail, 2024 (31) | UAE | None | HR | LR | HR | HR | LR | LR | LR | NA | LR | LR | LR |
| Moawd, 2022 (32) | Saudi Arabia | None | HR | LR | LR | LR | LR | LR | LR | HR | LR | LR | LR |
| Reddy, 2023 (33) | Saudi Arabia | None | HR | LR | HR | HR | LR | LR | LR | NA | LR | LR | LR |
| Yasmeen, 2025 (34) | Saudi Arabia | None | HR | LR | HR | HR | LR | LR | LR | NA | LR | LR | LR |
| Alasmari, 2023 a (16) | Saudi Arabia | None | HR | LR | HR | HR | LR | LR | NA | LR | LR | LR | LR |
| UPPER-MIDDLE INCOME COUNTRIES | | | | | | | | | | | | | |
| Al-Ardhi, 2022 (35) | Iraq | NR | HR | LR | HR | HR | LR | HR | LR | NA | LR | LR | LR |
| Qader, 2013 (36) | Iraq | None [f] | LR | LR | LR | HR | LR | HR | NA | HR | LR | LR | LR |
| Muhaidat, 2022 (37) | Jordan | None [g] | LR | LR | HR | HR | LR | HR | NA | LR | LR | LR | LR |
| Alghwiri, 2024 (38) | Jordan | None | HR | LR | HR | HR | LR | LR | NA | LR | LR | LR | LR |
| LOWER-MIDDLE INCOME COUNTRIES | | | | | | | | | | | | | |
| Abdelkhalik, 2023(39) | Egypt | NR | HR | LR | HR | HR | LR | LR | LR | HR | LR | LR | LR |
| Adly, 2020 (40) | Egypt | None | HR | LR | HR | HR | LR | HR | LR | NA | LR | LR | LR |
| Al Tehewy, 2015 (41) | Egypt | NR | HR | LR | LR | LR | LR | LR | LR | NA | LR | LR | LR |

| | | | | | | | | | | | | | |
|---|---|---|---|---|---|---|---|---|---|---|---|---|---|
| Ali Ibrahim, 2023(42) | Egypt | NR | HR | LR | HR | HR | LR | HR | LR | LR | LR | LR | LR |
| Amer, 2018 (43) | Egypt | None | HR | LR | HR | HR | LR | HR | LR | LR | LR | LR | LR |
| El-Gilany, 2011 (44) | Egypt | NR | HR | LR | LR | LR | LR | HR | LR | NA | LR | LR | LR |
| El-Gilany, 2013 (45) | Egypt | None | HR | LR | HR | HR | LR | HR | LR | NA | LR | LR | LR |
| El-Kawaly, 2016 (46) | Egypt | NR | HR | LR | HR | HR | LR | HR | LR | NA | LR | LR | LR |
| El-Rahman, 2014 (47) | Egypt | None | HR | LR | HR | HR | LR | HR | LR | LR | LR | LR | LR |
| Elsamahy, 2019 (48) | Egypt | NR | HR | LR | HR | HR | LR | HR | NA | HR | LR | LR | LR |
| Hassan, 2023a(49) | Egypt | None | HR | LR | HR | HR | LR | HR | NA | LR | LR | LR | LR |
| Ibrahim, 2019 (50) | Egypt | NR | HR | LR | HR | HR | LR | HR | LR | NA | LR | LR | LR |
| Ismail, 2018 (51) | Egypt | NR | LR | LR | HR | HR | LR | HR | LR | LR | LR | LR | LR |
| Khater, 2012 (52) | Egypt | None | HR | LR | HR | LR | LR | LR | LR | HR | LR | LR | LR |
| Mahmoued, 2014 (53) | Egypt | NR | LR | LR | HR | HR | LR | LR | LR | LR | LR | LR | LR |
| Makhlouf, 2000 (54) | Egypt | NR | LR | LR | HR | HR | LR | LR | LR | NA | LR | LR | LR |
| Mohamed, 2019 (55) | Egypt | NR | LR | LR | HR | HR | LR | LR | LR | NA | LR | LR | LR |
| Mohammed, 2021 (56) | Egypt | NR | HR | LR | HR | HR | LR | LR | LR | NA | LR | LR | LR |
| Mohsen, 2019 (57) | Egypt | None | LR | LR | LR | HR | LR | HR | NA | LR | LR | LR | LR |
| Naeem, 2020 (58) | Egypt | NR | HR | LR | HR | HR | LR | HR | LR | NA | LR | LR | LR |
| Rasheedy, 2021 (59) | Egypt | None | HR | LR | HR | HR | LR | HR | LR | NA | LR | LR | LR |
| Saleh, 2018 (60) | Egypt | NR | LR | LR | LR | HR | LR | HR | LR | LR | LR | LR | LR |
| Salman, 2024(62) | Egypt | NR | HR | LR | HR | HR | LR | HR | LR | LR | LR | LR | LR |
| Sharaf, 2008 (61) | Egypt | NR | LR | LR | LR | LR | LR | HR | NA | LR | LR | LR | LR |
| Tawfik, 2021(63) | Egypt | None | HR | LR | LR | LR | LR | HR | LR | NA | LR | LR | LR |
| Mahdi, 2017 (64) | Morocco | None | HR | LR | HR | HR | LR | HR | LR | HR | LR | LR | LR |
| Adnan, 2022 (65) | Pakistan | NR | HR | LR | HR | HR | LR | HR | HR | HR | LR | LR | LR |

| | | | | | | | | | | | | | |
|---|---|---|---|---|---|---|---|---|---|---|---|---|---|
| Anwar, 2023 (66) | Pakistan | None | LR | LR | HR | HR | LR | HR | LR | NA | LR | LR | LR |
| Bibi, 2023 (67) | Pakistan | None | HR | LR | HR | HR | LR | HR | LR | NA | LR | LR | LR |
| Gul, 2021 (68) | Pakistan | None | HR | LR | HR | HR | LR | HR | LR | LR | LR | LR | LR |
| Mehmood, 2016 (69) | Pakistan | NR | HR | LR | HR | HR | LR | HR | LR | NA | LR | LR | LR |
| Naseer, 2022 (70) | Pakistan | None | HR | HR | HR | HR | LR | HR | LR | NA | LR | LR | LR |
| Shahid, 2020 (72) | Pakistan | None | HR | LR | HR | HR | LR | HR | LR | LR | LR | LR | LR |
| Tariq, 2018 (73) | Pakistan | None | HR | LR | HR | HR | LR | HR | LR | NA | LR | LR | LR |
| Zulfiqar, 2019 (74) | Pakistan | None | HR | LR | HR | HR | LR | HR | NA | LR | LR | LR | LR |
| Nisar, 2024 (71) | Pakistan | None | HR | LR | HR | HR | LR | HR | LR | NA | LR | LR | LR |
| Badrasawi, 2022 (75) | Palestine | None | HR | LR | HR | LR | LR | LR | LR | LR | LR | LR | LR |
| Halaweh, 2016 a (76) | Palestine | NR | HR | LR | HR | HR | LR | LR | LR | NA | LR | LR | LR |
| Abbas, 2023 (77) | Lebanon | None | HR | LR | LR | HR | LR | LR | LR | NA | LR | LR | LR |
| Abd Elrahman, 2024 (78) | Egypt | None | HR | LR | HR | HR | LR | LR | NA | LR | LR | LR | LR |
| Abo Elyazed, 2025 (79) | Egypt | None | HR | LR | LR | HR | LR | LR | LR | NA | LR | LR | LR |
| Abdullah, 2025 (80) | Pakistan | None [h] | HR | LR | HR | HR | LR | LR | LR | LR | LR | LR | LR |
| Altaf, 2025 (81) | Pakistan | None | HR | LR | HR | HR | LR | LR | NA | LR | LR | LR | LR |
| Ammar, 2024 (82) | Pakistan | None | HR | LR | LR | HR | LR | LR | LR | NA | LR | LR | LR |
| Arshad, 2022 (83) | Pakistan | None | HR | LR | HR | HR | LR | HR | LR | LR | LR | LR | LR |
| Badrasawi, 2021 (84) | Palestine | None | HR | LR | LR | HR | LR | HR | LR | NA | LR | LR | LR |
| Battesha, 2022 (85) | Egypt | None | HR | LR | LR | HR | LR | LR | LR | NA | LR | LR | LR |
| Dwedar, 2025 (86) | Egypt | None | HR | LR | LR | HR | LR | LR | LR | NA | LR | LR | LR |
| Edarous, 2025 (87) | Egypt | None | HR | LR | HR | LR | LR | LR | LR | NA | LR | LR | LR |
| El Miedany, 2023 (88) | Egypt | None | LR | LR | HR | HR | LR | LR | LR | NA | LR | LR | LR |
| El Miedany, 2023a (89) | Egypt | NR | HR | LR | HR | HR | LR | LR | LR | NA | LR | LR | LR |

| | | | | | | | | | | | | | |
|---|---|---|---|---|---|---|---|---|---|---|---|---|---|
| Mohammed Zein El-Abdeen Mohamed, 2025 (90) | Egypt | None | HR | LR | HR | HR | LR | LR | NA | LR | LR | LR | LR |
| Eldegwey, 2025 (91) | Egypt | None | HR | LR | LR | HR | LR | HR | LR | NA | LR | LR | LR |
| Elgayar, 2025 (92) | Egypt | None | HR | LR | LR | HR | LR | LR | LR | NA | LR | LR | LR |
| Elsaid, 2024 (93) | Egypt | None | HR | HR | HR | HR | LR | LR | NA | LR | LR | LR | LR |
| Elshoura, 2025 (94) | Egypt | None [i] | HR | LR | HR | HR | LR | LR | LR | NA | LR | LR | LR |
| Fatima, 2022 (95) | Pakistan | None | HR | LR | LR | HR | LR | LR | LR | NA | LR | LR | LR |
| Ferhi, 2023 (96) | Tunisia | None | HR | LR | HR | HR | LR | LR | LR | NA | LR | LR | LR |
| Ghous, 2024 (97) | Pakistan | None | HR | LR | LR | HR | LR | LR | LR | NA | LR | LR | LR |
| Hassan Ahmed, 2024 (98) | Egypt | NR | HR | LR | HR | HR | LR | HR | NA | LR | LR | LR | LR |
| Hussein, 2024 (99) | Egypt | None | HR | HR | HR | HR | LR | LR | LR | NA | LR | LR | LR |
| Kandeel, 2024 (100) | Egypt | None | HR | LR | LR | HR | LR | LR | NA | LR | LR | LR | LR |
| Kaynat, 2025 (101) | Pakistan | None | HR | LR | HR | HR | LR | LR | NA | LR | LR | LR | LR |
| Khalil, 2024 (102) | Egypt | None | HR | LR | HR | HR | LR | HR | NA | LR | LR | LR | LR |
| Khallaf, 2025 (103) | Egypt | None | HR | LR | LR | HR | LR | LR | LR | NA | LR | LR | LR |
| Magtouf, 2023 (104) | Tunisia | None | HR | LR | LR | HR | LR | LR | LR | NA | LR | LR | LR |
| Mousa, 2016 (105) | Egypt | None | HR | LR | LR | HR | LR | LR | LR | NA | LR | LR | LR |
| Nazeer, 2025 (106) | Pakistan | NR | HR | LR | HR | HR | LR | HR | LR | LR | LR | LR | LR |
| Sayed, 2025 (107) | Egypt | None | HR | LR | HR | HR | LR | LR | LR | NA | LR | LR | LR |
| Waheed, 2024 (108) | Pakistan | None | HR | LR | HR | HR | LR | HR | LR | NA | LR | LR | LR |
| Zahra, 2023 (109) | Pakistan | None | LR | LR | HR | HR | LR | HR | LR | NA | LR | LR | LR |

LR= Low Risk of Bias; HR= High Risk of Bias; NA= Not Applicable.

a-The authors would like to thank the Deanship of Scientific Research at Umm Al-Qura University for supporting this work by Grant Code: 22UQU4280521DSR01.
b- The work is funded by grant number (15-MEd5221-10) from the National science, technology and innovation plan (Maarifah), the King Abdul-Aziz city for science and technology (Kast), Kingdom of Saudi Arabia.

c- The study was funded by the Ministry of Health, Riyadh, Kingdom of Saudi Arabia, as a part of the total fund provided for Older People Health Care Program in 2019 (approval number:22-26 M)
d-The authors extend their appreciation to the Deanship of Research and Graduate Studies at King Khalid University for funding this work through General Research Project under grant number GRP/74/46.
e- The authors extend their gratitude to the Deanship of Research and Graduate Studies at King Khalid University, KSA, for funding this work through a Small Research Group under grant number RGP. 1/117/45
f-This study was funded by the University Kebangsaan Malaysia Medical Centre Fundamental Research Grant, Code Number FF-017-2012 without which the study would not have been possible.
g- The study was funded by the Deanship of Academic Research at The University of Jordan (grant number Deanship of Academic Research, The University of Jordan 1517)
h-This work was funded by the Chulalongkorn University Thailand Graduate Scholarship Program for ASEAN or Non-ASEAN countries and supported by the Physiotherapy Department at Abasyn University, Pakistan.
i-The only conflict of interest is that Dr. Salwa Galal Moussa is an associate editor in the Egyptian Rheumatology and Rehabilitation journal where the articles is published

Notes:

Risk of Bias in Prevalence studies tool(110)

1. Was the study's target population a close representation of the national population in relation to relevant variables?
2. Was the sampling frame a true or close representation of the target population?
3. Was some form of random selection used to select the sample, OR was a census undertaken?
4. Was the likelihood of nonresponse bias minimal?
5. Were data collected directly from the subjects (as opposed to a proxy)?
6. Was an acceptable case definition used in the study?

7a. Was the study instrument that measured the parameter of interest (Risk of fall) shown to have validity and reliability?
7b. Was the study instrument that measured the parameter of interest (Fear of fall) shown to have validity and reliability?

8. Was the same mode of data collection used for all subjects?
9. Was the length of the shortest prevalence period for the parameter of interest appropriate?
10. Were the numerator(s) and denominator(s) for the parameter of interest appropriate?

Figure S1: Summary of the quality assessment of the included primary studies

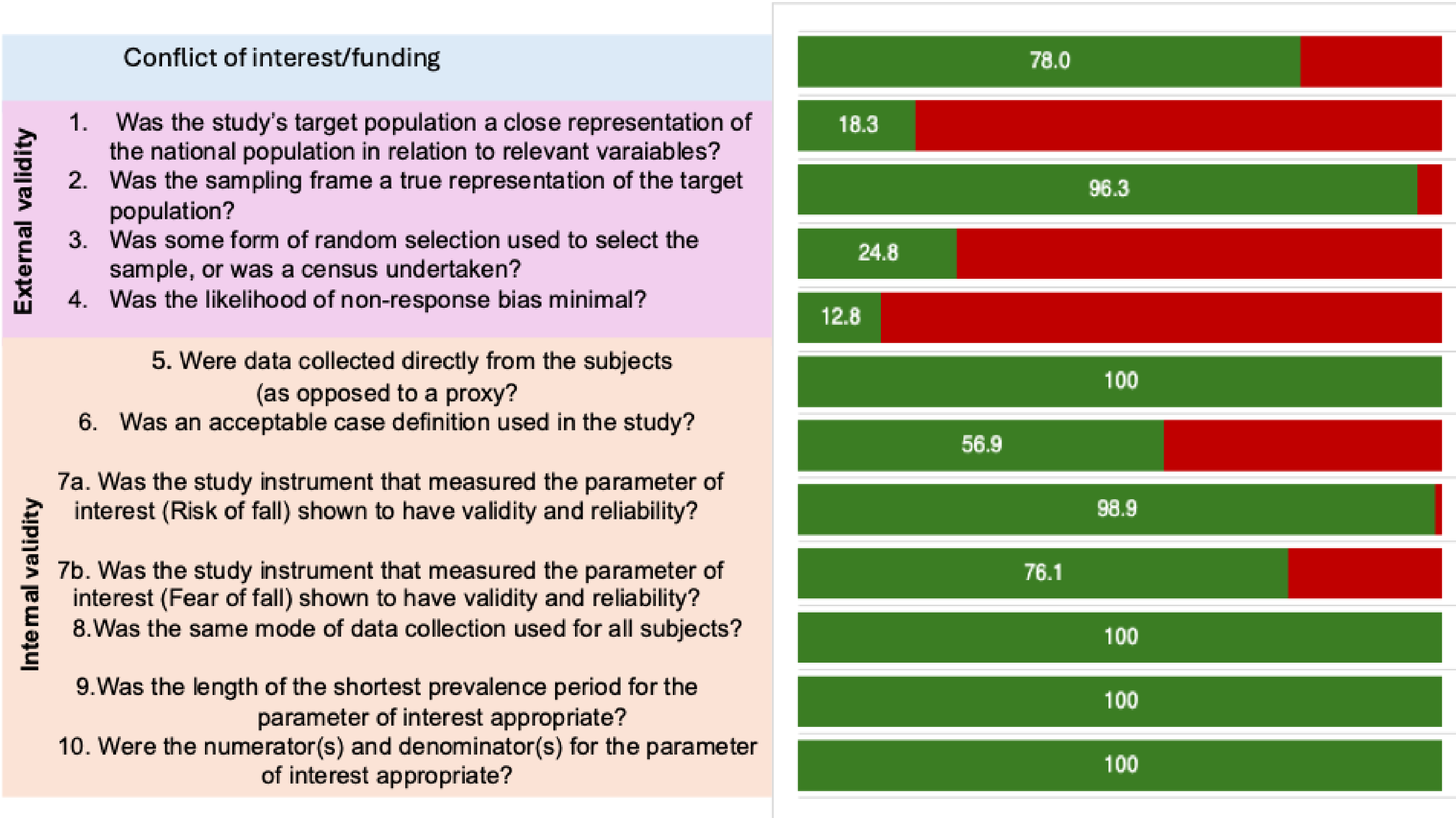

Figure S2: Meta-analysis of the mean score of fear of falling measured using standardized tests

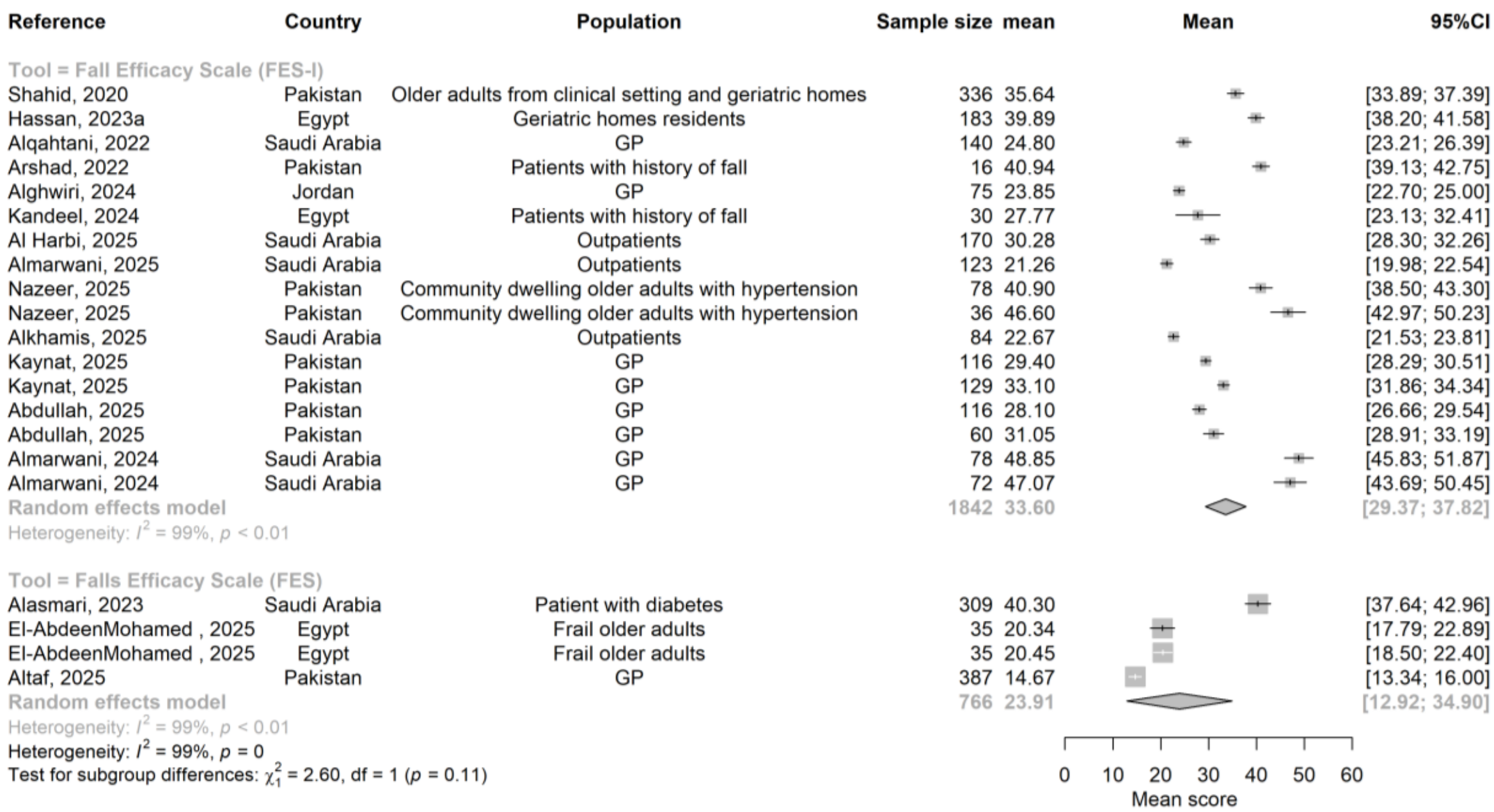

Table S4: Meta-analysis of the mean score of risk of falling measured using standardized tests

| Category | Number of datapoints | Sample Size | Range of mean scores | Pooled Estimate [95% CI] | Heterogeneity measures | | Q between subgroup tests (p-value) |
|---|---|---|---|---|---|---|---|
| | | | | | Q p-value | $I^2$(%) | |
| **Berg Balance Scale** | | | | | | | |
| Overall | 41 | 2439 | 0.71–53.65 | 40.06 [36.95–43.17] | 0.00 | 100.00 | NA |
| **Population** | | | | | | | |
| General population | 10 | 926 | 33.64–52.98 | 45.53 [41.98–49.07] | 0.00 | 99.80 | <0.0001 |
| Older adults with history of falls | 6 | 232 | 34.44–39.62 | 37.47 [35.80–39.14] | <0.01 | 97.60 | |
| Nursing homes residents | 4 | 174 | 33.30–39.60 | 34.48 [32.13–36.83] | 0.02 | 69.30 | |
| Outpatients | 14 | 794 | 35.00–53.65 | 44.42 [41.26–47.59] | <0.01 | 98.40 | |
| Patients with balance impairment | 2 | 64 | 38.63–39.78 | 39.11 [38.00–40.22] | 0.13 | 55.40 | |
| Chronic nonspecific neck pain | 1 | 93 | 47.85 | NA | NA | NA | |
| Community dwelling hypertensive | 2 | 114 | 0.71–11.22 | 5.96 [0–16.26] | <0.0001 | 100.00 | |
| Community dwelling diabetic | 2 | 42 | 32.05–32.57 | 32.40 [30.54–34.25] | 0.70 | 0.00 | |
| **Sex** | | | | | | | |
| Male/Female | 35 | 1975 | 32.05–53.65 | 41.23 [39.11–43.34] | 0.00 | 99.2 | 0.39 |
| Female | 6 | 464 | 0.71–52.98 | 33.49 [15.90–51.07] | 0.00 | 100 | |
| **Tinetti Assessment Tool** | | | | | | | |
| Overall | 9 | 239 | 15.50–23.65 | 20.01 [18.65–21.37] | <0.01 | 83.00 | NA |
| **Population** | | | | | | | |
| Rheumatoid arthritis | 2 | 40 | 15.50–21.30 | 19.08 [13.55-24.61] | 0.05 | 74.40 | 0.0008 |
| Elderly patients with balance and gait issues | 2 | 60 | 17.86–18.93 | 18.32 [17.28-19.36] | 0.27 | 16.70 | |
| Outpatients | 2 | 49 | 18.90–19.01 | 18.97 [18.13-19.81] | 0.9 | 0.00 | |
| Hospitalized | 2 | 90 | 20.31–23.65 | 22.24 [20.63-23.86] | 0.13 | 50.20 | |
| **Time up and go test** | | | | | | | |
| Overall | 66 | 4204 | 8.45–36.04 | 15.42 [14.23–16.61] | 0.00 | 98.00 | NA |
| **Population** | | | | | | | |
| General population | 18 | 1369 | 10.00–20.00 | 12.81 [12.03–13.58] | <0.01 | 95.40 | <0.0001 |
| Mixed outpatient or Geriatric residents/ non-fallers | 1 | 140 | 10.37 | NA | NA | NA | |
| Nursing homes residents | 4 | 258 | 12.22–15.22 | 14.31 [13.16–15.46] | <0.01 | 87.00 | |
| Cardiac surgery | 2 | 180 | 13.81–15.30 | 14.55 [13.09–16.01] | <0.01 | 91.40 | |
| Hospitalized | 5 | 296 | 17.57–33.66 | 24.13 [19.60–28.66] | <0.01 | 91.00 | |
| Rheumatoid arthritis | 2 | 40 | 18.40–24.00 | 20.14 [15.06–25.22] | 0.13 | 55.50 | |

| | | | | | | | |
|---|---|---|---|---|---|---|---|
| Outpatients | 21 | 1184 | 8.45–36.04 | 15.00 [12.62–17.37] | 0.00 | 98.80 | |
| Elderly patients with balance and gait issues | 2 | 60 | 18.93–19.22 | 19.14 [18.39–19.89] | 0.74 | 0.00 | |
| History of falls | 2 | 358 | 20.12–24.46 | 22.24 [17.99–26.49] | <0.01 | 95.00 | |
| Chronic nonspecific neck pain | 1 | 93 | 11.3 | NA | NA | NA | |
| Community dwelling hypertensive | 2 | 114 | 9.22–9.66 | 9.55 [8.93–10.16] | 0.54 | 0.00 | |
| Community dwelling diabetic | 2 | 42 | 21.31–22.90 | 22.27 [20.74–23.79] | 0.31 | 2.70 | |
| Heart failure | 1 | 20 | 20.6 | NA | NA | NA | |
| Obesity | 2 | 50 | 12.90–14.10 | 13.49 [12.3–14.66] | 0.03 | 77.70 | |
| **Sex** | | | | | | | |
| Male | 9 | 526 | 10.20–29.00 | 15.23 [11.71–18.75] | <0.01 | 96.00 | |
| Female | 17 | 1271 | 9.22–36.04 | 14.29 [12.06-16.53] | <0.01 | 95.70 | 0.50 |
| Male/Female | 40 | 2407 | 8.45–33.56 | 15.92 [14.38-17.45] | 0.00 | 98.90 | |

Table S5: Results of meta-regressions aimed at identifying associations and sources of between-study heterogeneity in the prevalence of older adults with risk of falling in MENA (Middle east and north Africa).

| | Number of datapoints | Total sample size | Univariable analyses | | | Unaccounted variability I2 |
|---|---|---|---|---|---|---|
| | | | OR (95% CI) | p value | LR test p value | |
| **Population type** | | | | | | |
| General Population | 10 | 1846 | 1·00 (ref) | .. | <0·0001 | 97.01% |
| Cardiac surgery | 2 | 180 | 0.55 (0.11–2.72) | 0.4642 | .. | .. |
| COVID-19 | 1 | 42 | 0.80 (0.09–7.25) | 0.8449 | .. | .. |
| Diabetic | 1 | 107 | 3.28 (0.38–28.58) | 0.282 | .. | .. |
| Inpatients | 4 | 841 | 0.82 (0.24–2.75) | 0.7458 | .. | .. |
| MSK pain | 1 | 71 | 1.99 (0.23–17.54) | 0.5337 | .. | .. |
| Nursing home residents | 5 | 577 | 0.36 (0.12–1.11) | 0.0752 | .. | .. |
| Outpatients | 9 | 1698 | 0.71 (0.27–1.83) | 0.4762 | .. | .. |
| **Sex** | | | | | | |
| Women | 3 | 459 | 1·00 (ref) | .. | <0·0001 | 97.43% |
| Men | 4 | 516 | 0.52 (0.09–2.84) | 0.4508 | .. | .. |
| Men & women | 26 | 4387 | 0.85 (0.22–3.28) | 0.8125 | .. | .. |
| **World Bank income levels** | | | | | | |
| High Income | 9 | 1515 | 1·00 (ref) | | <0·0001 | 97.26% |
| Upper-middle income | 1 | 107 | 3.16 (0.33–30.67) | 0.3201 | .. | .. |
| Lower-middle income | 23 | 3740 | 0.62 (0.27–1.45) | 0.2691 | .. | .. |
| **Assessment tool** | | | | | | |
| Berg Balance Scale | 11 | 1600 | 1·00 (ref) | .. | <0·0001 | 96.96% |
| Missouri Alliance Fall Risk Assessment Tool for Home Care (MAHC-10) | 6 | 1008 | 1.68 (0.57–4.92) | 0.3458 | .. | .. |
| Morse Fall Scale (MFS) | 7 | 1391 | 0.70 (0.25–1.96) | 0.4999 | .. | .. |
| Time up and go test (TUGT) | 9 | 1363 | 1.93 (0.74–4.99) | 0.1757 | .. | .. |
| **Age definition** | | | | | | |
| ≥60 | 28 | 4559 | 1·00 (ref) | .. | <0·0001 | 97.44% |
| ≥65 | 5 | 803 | 0.73 (0.24–2.19) | 0.5773 | .. | .. |
| **Sample size** | | | | | | |
| ≥200 | 8 | 2527 | 1·00 (ref) | .. | <0·0001 | 97.41% |
| <200 | 25 | 2835 | 1.32 (0.53–3.25) | 0.5497 | .. | .. |
| **Response rate** | | | | | | |
| ≥80% | 5 | 732 | 1·00 (ref) | .. | <0·0001 | 97.13% |
| 70 | 1 | 81 | 0.62 (0.06–6.28) | 0.6875 | | |
| Not reported | 27 | 4549 | 2.74 (0.99–7.60) | 0.0528 | .. | .. |
| **Sampling methodology** | | | | | | |
| All target population or random | 5 | 1415 | 1·00 (ref) | .. | <0·0001 | 97.39% |
| Non-probability or unclear | 28 | 3947 | 0.89 (0.30–2.63) | 0.8352 | .. | .. |
| **Year of data collection** | 33 | 5362 | 0.97 (0.86–1.08) | 0.5783 | <0·0001 | 97.40% |

*nothing was significant in the univariable analysis

Table S6: Univariable meta-regression of individual Risk of Bias Tool for Prevalence Studies items and prevalence of higher fall risk

| Hoy item | Risk-of-bias domain | LR data points | HR data points | β (HR vs LR) | 95% CI | p-value |
|---|---|---|---|---|---|---|
| 1 | National representativeness of the study population | 9 | 24 | −0.570 | −1.426–0.287 | 0.192 |
| 2 | Representativeness of the sampling frame | 30 | 3 | −0.862 | −2.207–0.483 | 0.209 |
| 3 | Random selection or census | 5 | 28 | −0.115 | −1.199–0.969 | 0.835 |
| 4 | Minimal risk of non-response bias | 6 | 27 | 0.858 | −0.114–1.829 | 0.084 |
| 5 | Data collected directly from participants/records | 33 | 0 | Not estimable | — | — |
| 6 | Acceptable definition of the outcome | 9 | 24 | 0.197 | −0.683–1.077 | 0.661 |
| 7 | Valid and reliable measurement instrument | 33 | 0 | Not estimable | — | — |
| 8 | Same mode of data collection used for all participants | 33 | 0 | Not estimable | — | — |
| 9 | Appropriate prevalence period | 33 | 0 | Not estimable | — | — |
| 10 | Appropriate numerator and denominator | 33 | 0 | Not estimable | — | — |

Risk of Bias Tool for Prevalence Studies developed by Hoy et al. (2012)(110)
No meta-regression for items 5, 7, 8, 9, or 10. There was no HR/LR contrast, so the model cannot assess their influence.
**Abbreviations:** LR, low risk of bias; HR, high risk of bias; CI, confidence interval.

Table S7: Results of meta-regressions aimed at identifying associations and sources of between-study heterogeneity in the prevalence of older adults with fear of falling in MENA (Middle east and north Africa).

| | Number of datapoints | Total sample size | Univariable analyses OR (95% CI) | p value | LR test p value | Unaccounted variability I2 |
|---|---|---|---|---|---|---|
| **Population type** | | | | | | |
| General Population | 15 | 1814 | 1·00 (ref) | .. | <0·0001 | 98.17% |
| Elderly from clinical setting and geriatric homes | 3 | 1008 | 0.76 (0.10–5.56) | 0.7870 | .. | .. |
| Geriatric homes residents | 1 | 208 | 0.80 (0.03–20.45) | 0.8918 | .. | .. |
| Outpatient or inpatient | 1 | 84 | 4.35 (0.16–116.06) | 0.3802 | .. | .. |
| Outpatients | 5 | 611 | 0.40 (0.08–2.067) | 0.2756 | | |
| Patient with history of fall | 5 | 342 | 1.91 (0.36–10.05) | 0.4472 | | |
| Patients with eye problem | 1 | 77 | 9.00 (0.32–251.24) | 0.1959 | | |
| **Sex** | | | | | | |
| Women | 10 | 1153 | 1·00 (ref) | .. | <0·0001 | 98.38% |
| Men | 4 | 425 | 0.24 (0.03–1.71) | 0.1533 | .. | .. |
| Men and Women | 17 | 2566 | 0.72 (0.19–2.76) | 0.6328 | .. | .. |
| **World Bank income levels** | | | | | | |
| High Income | 7 | 573 | 1·00 (ref) | | <0·0001 | 98.56% |
| Low -middle income | 24 | 3571 | 0.57 (0.13–2.56) | 0.4634 | .. | .. |
| **Assessment Tool** | | | | | | |
| Falls Efficacy Scale-International (FES-I) | 13 | 1671 | 1·00 (ref) | | <0·0001 | 98.28% |
| Falls Efficacy Scale-International (FES-I) - Arabic version | 6 | 600 | 0.29 (0.06–1.47) | 0.1351 | | |
| Modified Falls Efficacy Scale (MFES) | 9 | 1573 | 1.42 (0.34–5.87) | 0.628 | .. | .. |
| Short fall Efficacy Scale-international (SFES-I) | 3 | 300 | 0.76 (0.09–6.06) | 0.7921 | .. | .. |
| **Age definition** | | | | | | |
| ≥60 | 20 | 2205 | 1·00 (ref) | | <0·0001 | 98.47% |
| ≥65 | 11 | 1939 | 0.54 (0.15–1.94) | 0.3445 | | |
| **Sample size** | | | | | | |
| <200 | 24 | 2164 | 1·00 (ref) | | <0·0001 | 98.53% |
| ≥200 | 7 | 1980 | 0.97 (0.22–4.21) | 0.9625 | | |
| **Response rate** | | | | | | |
| >80% | 10 | 1816 | 1·00 (ref) | | <0·0001 | 98.36% |
| Not Reported | 21 | 2328 | 3.09 (0.87–10.87) | 0.0797 | | |
| **Sampling methodology** | | | | | | |
| All target population or random | 7 | 956 | 1·00 (ref) | | <0·0001 | 98.51% |
| Non-probability or unclear | 24 | 3188 | 0.83 (0.17–3.64) | 0.8003 | | |
| **Year of data collection** | 31 | 4144 | 1.00 (0.84–1.20) | 0.9608 | <0·0001 | 98.52% |

*nothing was significant in the univariable analysis

Table S8. Univariable meta-regression of individual Risk of Bias Tool for Prevalence Studies items and prevalence of fear of falling

| Hoy item | Risk-of-bias domain | LR data points | HR data points | β (HR vs LR) | 95% CI | p-value |
|---|---|---|---|---|---|---|
| 1 | National representativeness of the study population | 5 | 26 | −0.260 | −1.928–1.408 | 0.760 |
| 2 | Representativeness of the sampling frame | 31 | 0 | Not estimable | — | — |
| 3 | Random selection or census | 7 | 24 | −0.192 | −1.677–1.293 | 0.800 |
| 4 | Minimal risk of non-response bias | 7 | 24 | 1.231 | −0.179–2.641 | 0.087 |
| 5 | Data collected directly from participants/records | 31 | 0 | Not estimable | — | — |
| 6 | Acceptable definition of the outcome | 18 | 13 | 0.136 | −1.119–1.391 | 0.832 |
| 7 | Valid and reliable measurement instrument | 31 | 0 | Not estimable | — | — |
| 8 | Same mode of data collection used for all participants | 31 | 0 | Not estimable | — | — |
| 9 | Appropriate prevalence period | 31 | 0 | Not estimable | — | — |
| 10 | Appropriate numerator and denominator | 31 | 0 | Not estimable | — | — |

Risk of Bias Tool for Prevalence Studies developed by Hoy et al. (2012)(110)
No meta-regression for items 2, 5, 7, 8, 9, or 10. There was no HR/LR contrast, so the model cannot assess their influence
**Abbreviations:** LR, low risk of bias; HR, high risk of bias; CI, confidence interval.

Figure S3: doi plot assessing publication bias in studies reporting risk of falls and fear of falling

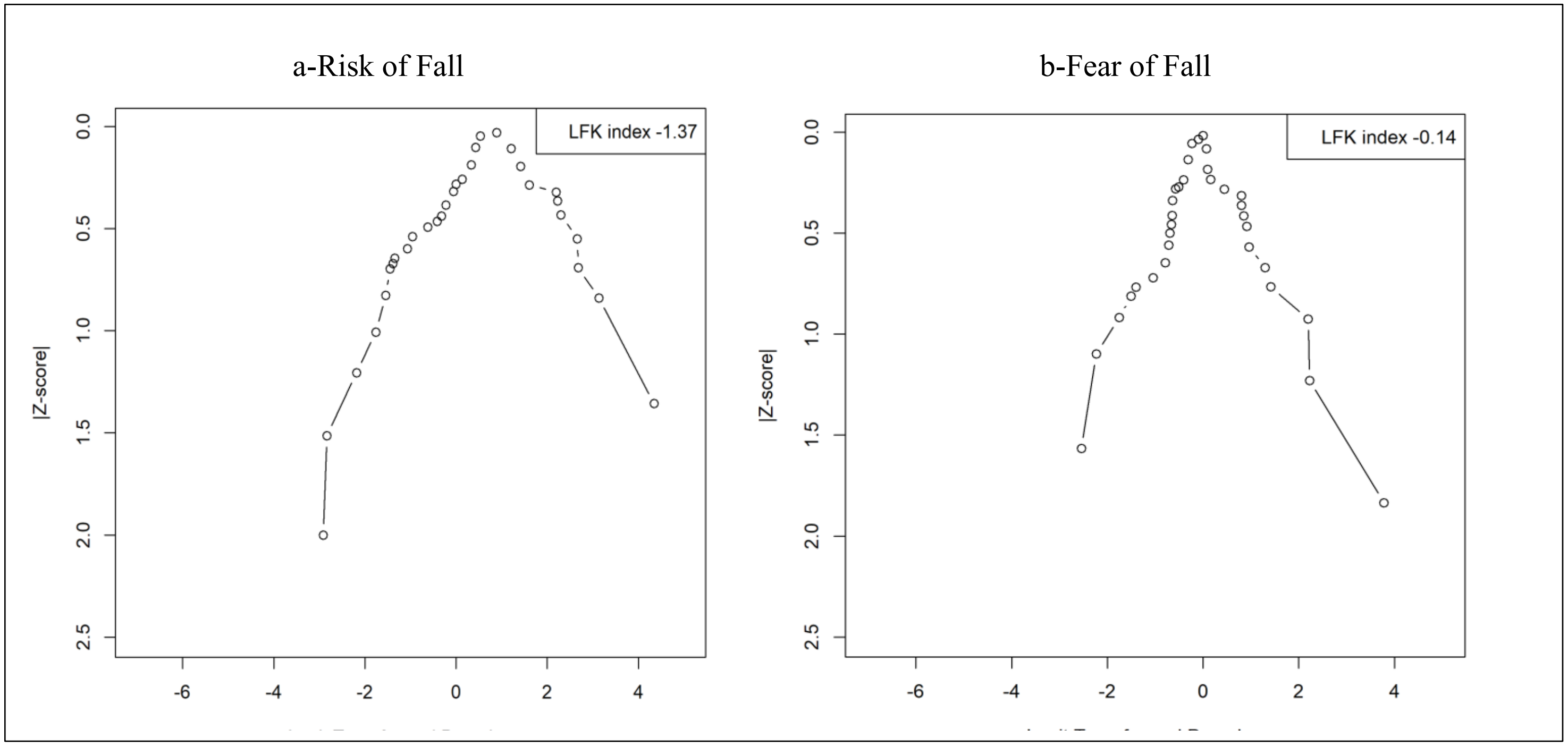


Note: doi plot shows normal-quantile (Z-score) against logit transformation of prevalence (X-axis), and LFK index to assess publication bias by visualizing asymmetry and quantifying potential asymmetry of study effects (111). There was no evidence of publication bias for fear of falling (LFK index=-0.14) while the LFK for risk of falling was -1.37 indicating asymmetry.

## Reference list:


1. Abdelhafez BMS, Serry ZMH, Elbanna RHM. Modified Pilates versus Conventional Balance on Gait Training Performance and Functional Status in Elderly. The Egyptian Journal of Hospital Medicine. 2023;90(1):1387-92.
2. Alabdullgader A, Rabbani U. Prevalence and Risk Factors of Falls Among the Elderly in Unaizah City, Saudi Arabia. Sultan Qaboos University medical journal. 2021;21(1):e86-e93.
3. Alharbi AA. Comparison of three fall risk assessment tools in community-dwelling Saudi elderlies: A prospective study. 2023.
4. Alshammari SA, Alhassan AM, Aldawsari MA, Bazuhair FO, Alotaibi FK, Aldakhil AA, et al. Falls among elderly and its relation with their health problems and surrounding environmental factors in Riyadh. Journal of family & community medicine. 2018;25(1):29-34.
5. Alshehri FH. The effect of COVID-19 on the risk of falling in old age adult patients: a cross-sectional study. European Review for Medical & Pharmacological Sciences. 2024;28(2).
6. Petrinec A, Dowell JA, APRN-CNP P. The Relationship Between Individual Perception of Perceived Susceptibility of Fall Risk and Fall Risk Level among Elderly Adult Patients Admitted in Acute Care Setting in a Saudi Arabian Hospital (KSFAH).
7. Abd El-Kafy EM, Alayat MS, Subahi MS, Badghish MS. C-Mill Virtual Reality/Augmented Reality Treadmill Training for Reducing Risk of Fall in the Elderly: A Randomized Controlled Trial. Games for Health Journal. 2024.
8. Alzaabi HS, Walton LM, Arumugam A. Association between demographic characteristics, lower limb range of motion, functional performance, ability to dual task, quality of life and risk of falls in older adults of the United Arab Emirates-A cross-sectional study. Heliyon. 2022;8(2):e08869.
9. Abd Elkader SM, Al-Shenqiti AM, El-Gohary TM. Timed Up and Go and Five Times Sit to Stand Test among community ambulant, overweight, obese Saudi elderly population. Journal of the Pakistan Medical Association. 2022;72(7):1306-10.
10. Abdelaal AA, El-Shamy SM. Effect of lower body positive pressure aerobic training on fall risk in patients with diabetic polyneuropathy: randomized controlled trial. European Journal of Physical and Rehabilitation Medicine. 2022;58(1):33-42.
11. Al Harbi RA, Omar MTA, Elkader S, Alhammad SA, Gwada RFM. Factors associated with fear of falling among Saudi community-dwelling older adults: A cross-sectional study. Medicine (Baltimore). 2025;104(24):e42864.
12. Alahmari KA, Reddy RS. Biomechanical analysis of limits of stability using computerized posturography: correlations with functional mobility in elderly individuals with hip osteoarthritis - a cross-sectional study. Frontiers in Bioengineering and Biotechnology. 2024;12.
13. Al-Amoud MM, Omar DI, Almashjary EN, Alomary SA. Morbidity profile among older people at primary health care centers in Saudi Arabia during the period 2012-2020. Saudi Med J. 2023;44(1):45-56.
14. Alamri SH, Ghamri RA, Alshehri WJ, Alhuthayli RS, Alamoudi NM, Alnufaei RD, et al. Falls and correlations among community-dwelling older adults: A Cross-sectional study in Jeddah, Saudi Arabia. Pakistan Journal of Medical Sciences. 2023;39(1).
15. Alanazi A, Salih S. Fall Prevalence and Associated Risk Factors Among the Elderly Population in Tabuk City, Saudi Arabia: A Cross-Sectional Study 2023. Cureus. 2023;15(9):e45317.
16. Alasmari RS, Hassani HA, Almalky NA, Bokhari AF, Al Zahrani A, Hafez AA. Risk factors for fall among the elderly with diabetes mellitus type 2 in Jeddah, Saudi Arabia, 2022: a cross-sectional study. Ann Med Surg (Lond). 2023;85(3):412-7.
17. Alharbi AA, Al Amer HS, Albalwi AA, Muthaffar MY, Alshehre YM, Albalawi HF, et al. Cross-Cultural Adaptation and Psychometric Properties of the Arabic Version of the Fall Risk Questionnaire. Int J Environ Res Public Health. 2023;20(8).

18. Alharbi AA, Albalwi AA, Al Amer HS, Alamrani SA, Albalawi HF, Alrashedi FD, et al. Nationwide assessment of fall risk and protective factors among older adults in Saudi Arabia. PLoS One. 2025;20(9):e0332900.
19. AlHarkan KS, Alsousi S, AlMishqab M, Alawami M, Almearaj J, Alhashim H, et al. Associations between polypharmacy and potentially inappropriate medications with risk of falls among the elderly in Saudi Arabia. Bmc Geriatrics. 2023;23(1).
20. Ali ARH, Aljohani MM, Alkhamees NH, Al-Shenqiti A. Autumnal Hazards: A Study of Falls among the Elderly in the Madinah Region—Prevalence, Risk Factors, and Consequences. 2024.
21. Aljarbou HM, Almoajel AM, Althomali MM, Almutairi KM. Risk and Preventive Measures Among Older Adults in Nursing Homes in Saudi Arabia: An Exploratory Study on Falls. Healthcare (Basel). 2025;13(3).
22. Alkhamis BA, Elrefaey BH, Almohiza MA, Alahmari KA, Alshahrani MS, Alnakhli HH, et al. Cognition, fear, and falls: psychological predictors of balance impairment in community-dwelling older adults. Frontiers in Psychiatry. 2025;16:1610894.
23. Almarwani M, Alosaimi B, Vincenzo JL. Cross-cultural adaptation and validation of the Arabic version of the Stay Independent Brochure as part of the CDC's STEADI initiative among community-dwelling older adults. BMC Geriatr. 2024;24(1):997.
24. Almarwani M. Moving the needle on implementing fall prevention programs in Saudi Arabia: assessing knowledge and perceptions of fall risk among community-dwelling older women. Bmc Geriatrics. 2024;24(1).
25. Almarwani MM, Alharbi WM, Alkeridy WA. Cultural adaptation and validation of the Arabic short version of the Iconographical Falls Efficacy Scale (Icon-FES): Assessing concern about falling among older adults. Aging Clin Exp Res. 2025;37(1):37.
26. Alqahtani BA, Alhowimel AS, Alshehri MM, Alqahtani MA, Almuhaysh AA, Alshakarah AO, et al. Cross-Cultural Adaptation and Validation of the Arabic Version of the Mini-BESTest among Community-Dwelling Older Adults in Saudi Arabia. Healthcare (Basel). 2022;10(10).
27. AlQahtani M. Impact of Wii Fit Training (WFT) and Reactive Balance Training (RBT) for in Elder Age Population: A Comparative Analysis. Journal of Pharmacy and Bioallied Sciences. 2024;16:S452-S5.
28. Alshehri SHS, Reddy RS, Almohiza MA, Alshahrani MS, Alkhamis BA, Alnakhli HH, et al. Influence of cervical muscle strength and pain severity on functional balance and limits of stability in elderly individuals with chronic nonspecific neck pain: a cross-sectional study. Bmc Geriatrics. 2025;25(1).
29. El-Bagalaty AE, Mohamed ME, Abdelraouf OR, Ghafar MAA, Abdelaal AK, Abdelgalil AA, et al. Balance and Fall Risk Assessment in Community-Dwelling Older Adults after Recovery from COVID-19: A Cross-Sectional Study. Sports. 2023;11(2).
30. Hasan S, Chevidikunnan MF, Khan F. Reliability and validity of the Arabic version of the modified falls efficacy scale. Disability and rehabilitation. 2024;46(4):793-801.
31. Ismail RM, Thomas D, Jagdale R. Influence of medications on fall risk assessment in maintenance hemodialysis patients: a cross-sectional study. International journal of critical illness and injury science. 2024;14(1):32-6.
32. Moawd SA, Nambi G, Alrawaili SM, Abdelbasset WK, Aboelmagd F, Elsayed SH. Analyzing the influence of the combination of monochromatic infrared energy and tai chi exercise improve balance in community-dwelling older adults with lower-extremity disease: a double-blinded randomized controlled study. European Review for Medical and Pharmacological Sciences. 2022;26(21):7788-96.
33. Reddy RS, Alkhamis BA, Kirmani JA, Uddin S, Ahamed WM, Ahmad F, et al. Age-Related Decline in Cervical Proprioception and Its Correlation with Functional Mobility and Limits of Stability Assessed Using Computerized Posturography: A Cross-Sectional Study Comparing Older (65+ Years) and Younger Adults. Healthcare. 2023;11(13).
34. Yasmeen A, Syed MH, Meraya AM, Alqahtani SS, Syed NK, Alfaifi AJ, et al. Fall Risk and Knowledge of Fall-Risk-Increasing Drugs Among Saudi Older Adults. Healthcare (Basel). 2025;13(13).

35. Al-Ardhi IS, Atiyah HH. Association Between Risk of Falls and Body Mass Index Among Elderly Diabetic Patients. Pakistan Journal of Medical & Health Sciences. 2022;16(03):1014-.
36. Abdal Qader MA, Amin RM, Shah SA, Isa ZM, Latif KA, Ghazi HF. Psychological risk factors associated with falls among elderly people in Baghdad city, Iraq. Open Journal of Preventive Medicine. 2013;3(07):441-5.
37. Muhaidat J, Al-Khlaifat L, Al-Yahya E, Mohammad M, Okasheh R, Qutishat D, et al. Factors Associated with Physical Activity in Jordanian Older People. Activities, Adaptation & Aging. 2022:1-18.
38. Alghwiri AA, Obeidat FS, Al-Jaghbeer J, Abuatiq RA, Whitney SL. Using an Arabic Version of the Life-Space Assessment to Evaluate How Gait Speed and Gender Predict Mobility Restrictions among Older Adults. Medicina-Lithuania. 2024;60(3).
39. Abdelkhalik AA. Assessment of elderly awareness regarding Balance Disorders and Falls Prevention. Helwan International Journal for Nursing Research and Practice. 2023;2(2):145-59.
40. Adly NN, Abd-El-Gawad WM, Abou-Hashem RM. Relationship between malnutrition and different fall risk assessment tools in a geriatric in-patient unit. Aging clinical and experimental research. 2020;32(7):1279-87.
41. al Tehewy MM, Amin GE, Nassar NW. A Study of Rate and Predictors of Fall Among Elderly Patients in a University Hospital. Journal of Patient Safety. 2015;11(4):210-4.
42. Ibrahim MAA, Ahmed AE-sH. Relationship between Visual Functioning, Balance, and Fear of Falling among Community-dwelling seniors with Cataract. Egyptian Journal of Health Care. 2023;14(4):807-25.
43. Amer MS, Abdellah AF, Elsayed ES. Fear of falls among nursing homes residents and community dwelling elderly. The Egyptian Journal of Hospital Medicine. 2018;73(5):6785-8.
44. El-Gilany A-H, Hatata E-s, Soliman S, Refaat R. Falls among elderly persons living in a rural community setting, Egypt. ME-JAA. 2011
6(5):18.
45. El-Gilany A-H, Hatata E-s, Soliman SM, Refaat R. Prevention of recurrent falls in elderly: a pre-post intervention study in a rural community, Egypt. International Journal of Collaborative Research on Internal Medicine & Public Health. 2013;5(4):0-.
46. El-Kawaly WH, Amer MS, Hamza SA, Arif ER. Fracture risk assessment in geriatric homes in Egypt. Indian J Med Res Pharmaceutical Sci. 2016;3(10):4-14.
47. Khattab Abd El-Rahman S. Impact of Balance Disorders on the Health Outcomes of Elderly People in Damanhur-Egypt. Journal of High Institute of Public Health. 2014;44(1):41-7.
48. Elsamahy EA, Ebtesam MW, Elsayed S. Risk Factors for fall among Elderly in a Rural Community in El-Monofya Governorate. Saudi Journal of Nursing and Health Care. 2019;2(1):1-14.
49. Hassan BA-A, Soliman A, El-Sayed MS, Ahmed KS, Abdelhakiem NM, El-Deen HAB, et al. Correlation Between Dyspnea, Fear of Falling and Quality of Life in Geriatric Population in Nile Delta Region, Egypt. International Journal. 2023;10(4):1628-36.
50. Ibrahim HM, Aly SE. Impact of Strengthening and Balance Training Exercise on Fall Risk among Elderly at Assiut Geriatric Clubs. 2019.
51. Ismail GM, Fahim HI, Bakr I, Wassif GO, Hamza SA. Risk of falls and Effect of a Health Education Program in Prevention of Falls among Elderly in Geriatric Homes in Cairo, Egypt. The Egyptian Journal of Geriatrics and Gerontology. 2018;5(2):1-7.
52. Khater MS, Mousa SM. Predicting falls among Egyptian nursing home residents: A 1-year longitudinal study. Journal of Clinical Gerontology and Geriatrics. 2012;3(2):73-6.
53. Mahmoued SB, Abd Allah ES, El-Naggar SAE-M. Prevalence of Falling among Elderly at Home in Rural Area of Zagazig District. Zagazig Nursing Journal. 2014;10(2):18-35.
54. Makhlouf MM, Ayoub AI. Falls among institutionalized elderly in Alexandria. J Egypt Public Health Assoc. 2000;75(5-6):507-28.

55. Mohamed AA, El-Shamaa ET, Mohamed JAE-R. Assess the effect of exercises program on balance and prevention of recurrent falling among elderly people. Minia Scientific Nursing Journal. 2019;6(1):63-70.
56. Mohammed RF, Alagamy ZG, El-saidy TM. Detection of the modifiable and Non-Modifiable risk factors and the adverse consequences of falling among elderly people. Assiut Scientific Nursing Journal. 2021;9(24):166-78.
57. Mohsen MM, Sabola NE-S, Abd El-Salam EA, Matar HL. Effect of Environmental Home Modification on Falling Risk Reduction among Elderly in a Rural Community. 2019.
58. Naeem EH, Mostafa NS, El-Said SMS. Prevalence of frailty among elderly patients attending primary health care centers in sixth of October city. EJGG. 2020;5(2):5-11.
59. Rasheedy D, El-Kawaly WH. The Cumulative Impact of Sarcopenia, Frailty, Malnutrition, and Cachexia on Other Geriatric Syndromes in Hospitalized Elderly. Electronic Journal of General Medicine. 2021;18(2).
60. Saleh NMH, Ibrahim HS, Mohamed HNAE-A, El-Gilany A-H. Predictors of fear of falling among community dwelling older adults in Mansoura City, Egypt. International Journal of Nursing Didactics. 2018;8(08):63-71.
61. Sharaf AY, Ibrahim HS. Physical and psychosocial correlates of fear of falling among older adults in assisted living facilities. Journal of Gerontological nursing. 2008;34(12):27-35.
62. Saad M. Risk Factors of Falling among Elderly People in Suez Canal University Hospitals. Trends in Nursing and Health Care Journal. 2024;8(1):65-85.
63. Tawfik HM, Desouki RR, Singab HA, Hamza SA, El Said S. Multidimentional Preoperative Frailty Assessment and Postoperative Complication Risk in Egyptian Geriatric Patients Undergoing Elective Cardiac Surgery. Journal of Alzheimer's Disease. 2021;82(1):391-9.
64. Mahdi MA, Erraoui M, Ngeuleu A, Tahiri L, Rkain H, Allali F. Falls, fear of falling and risk of falls in patients with rheumatoid arthritis: prevalence and associated factors. Pan African Medical Journal. 2017;28.
65. Adnan M, Anjum H, Adnan H, Ayesha HS, Alam W, Umar S, et al. Effectiveness of Physical Activity in Geriatric Population to Decrease the Risk of Fall a Randomized Control Trial. Pakistan Journal of Medical & Health Sciences. 2022;16(12):589-.
66. Anwar AW, F; Javaid, A; Zahoor, I. A; Malik, A. N & Abbas, R. Normative Values of Berg Balance Scale and Timed Up and Go Test in Elderly Females; A Descriptive Study : Berg Balance Scale in Elderly Females. The Healer Journal of Physiotherapy and Rehabilitation Sciences. 2023;3(6):617-26.
67. Bibi R, Yan Z, Anwar N, Zeb A, Mian N. Assessment of Fall-Associated Risk Factors in the Muslim Community-Dwelling Elders of Peshawar, Khyber Pakhtunkhwa, Pakistan. Preprintorg. 2023.
68. Gul S. Association between risk of fall and physical activity among the elderly population of police colony, Peshawar. Rehman Journal of Health Sciences. 2021;3(2):97-103.
69. Mariam M, Mushtaq M, Ansa N. Prevalence of risk of fall in community dwelling Pakistani male population over the age of 65 years. J Nov Physiother. 2016;5:2165e7025.
70. Naseer B, Dastgir H, Sadiq A, Salik S, Abid N, Tayyab M. Factors Associated with Risk of Fall in Elderly Population: Risk of Fall in Elderly Population. The Healer Journal of Physiotherapy and Rehabilitation Sciences. 2022;2(3):221-31.
71. Nisar A, Saleem M, Raza MH, Riaz H, Amjad F, Arshad A, et al. Assessment of Fall Risk and its Association with Frailty among Elderly: Fall Risk and its Association with Frailty. THE THERAPIST (Journal of Therapies & Rehabilitation Sciences). 2024:26-31.
72. Shahid S, Tariq MI, Ramzan T. Correlation of fall efficacy scale and Hendrich fall risk model in elder population of Rawalpindi-Islamabad. JPMA The Journal of the Pakistan Medical Association. 2020;70(11):1938-40.
73. Tariq K, Aleem F, Zia A, Jamil M. Pain characteristics and balance confidence in relation to fall risk in older adults with musculoskeletal pain. Rawal Medical Journal. 2018;43(3):553-7.

74. Zulfiqar F, Bashir MS, Noor R, Ahmad A, Gilani SA, Chugtai AS. Fear of fall among geriatric population. Rawal Medical Journal. 2019;44(1):64-6.
75. Badrasawi M, Hamdan M, Vanoh D, Zidan S, ALsaied T, Muhtaseb TB. Predictors of fear of falling among community-dwelling older adults: Cross-sectional study from Palestine. PLoS one. 2022;17(11):e0276967.
76. Halaweh H, Willen C, Grimby-Ekman A, Svantesson U. Physical functioning and fall-related efficacy among community-dwelling elderly people. European Journal of Physiotherapy. 2016;18(1):11-7.
77. Abbas RL, Saab IM, Al-Sharif HK, Naja N, El-Khatib A. Effect of Adding Motorized Cycle Ergometer Over Exercise Training on Balance in Older Adults with Dementia: A Randomized Controlled Trial. Experimental Aging Research. 2023;49(2):100-11.
78. Abd Elrahman AAE, Elsayed S, Ahmed H, Mouawad E. Vision Related Quality of Life and Fear of Falls among Elderly Patients with Macular Degeneration. Mansoura Nursing Journal. 2024;11(2):287-306.
79. Abo Elyazed TI, Alzorqany ASAF, Taha EMI. Effect of Eccentric Exercises Versus Core Stability Exercises on Falling Risk and Functional Capacity in The Elderly A Randomized Control Study. Delta University Scientific Journal. 2025;8(1):187-97.
80. Abdullah, Mathiyakom W, Asai T, Ali I, Ullah S, Tantisuwat A. Comparison of balance, functional capacity, and quality of life between older adults with lower and higher levels of physical activity. BMC GERIATRICS. 2025;25(1).
81. Altaf S, Malmir K, Syed J, Aftab A, Tariq H, Olyaei GR, et al. Prevalence and Impact of Probable Sarcopenia in Community-Dwelling Older Adults in Pakistan. Ageing International. 2025;50(2):1-18.
82. Ammar M, Amjad I, Nisar M, Ghoneim OSA. Effects of board games on balance in association with cognition in community-dwelling older adults. Journal of Musculoskeletal Surgery and Research. 2024;8(3):256-63.
83. Arshad N, Shabbir M, Hanif M. Use of berg balance scale to prevent fall in geriatric patients. Rawal Medical Journal. 2022;47(4):982-5.
84. Badrasawi MM, Natour NO, Jaradat A. Factors associated with history of fall among older people in southern West Bank. Journal of Gerontology and Geriatrics. 2021;69(3):155-63.
85. Battesha HHM, Wadee AN, Shafeek MM, Tawfick AM, Ibrahim HM. Maze Control Training on Kinesthetic Awareness in Patients with Stroke: A Randomized Controlled Trial. REHABILITATION RESEARCH AND PRACTICE. 2022;2022.
86. Dwedar HSEA, Elnahas NGM, Gendia WMI, Ali SM. Effect of Otago Exercises on Risk of Fall in Patients with Type 2 Diabetes Mellitus. The Egyptian Journal of Hospital Medicine (January 2025).98:1188-94.
87. Edarous DH, Fahim HI, Momen M, Shaat AAE, Manzour AF. Epidemiology of age-related macular degeneration among elderly in geriatric homes, East Cairo, Egypt. BMC Public Health. 2025;25(1):2495.
88. El Miedany Y, El Gaafary M, Gadallah N, Mahran S, Fathi N, Abu-Zaid MH, et al. Incidence and geographic characteristics of the population with osteoporotic hip fracture in Egypt- by the Egyptian Academy of Bone Health. Arch Osteoporos. 2023;18(1):115.
89. El Miedany Y, El Gaafary M, Gadallah N, Mahran S, Hassan W, Fathi N, et al. Targeted optimum care approach for osteoporotic fragility fractures: tailored strategy based on risk stratification to reduce incidents of falls-an initiative by the Egyptian Academy of bone health based on the FLS national register. Arch Osteoporos. 2023;18(1):139.
90. Mohamed Zein El-AbdeenMohamed D, Noshy Abd El-Aziz Mohamed H, Hassan Abd Elhameed S. Effect of a home-based physical rehabilitation program via virtual reality on the functional outcomes of frail older adults: a quasi-experimental study. Sci Rep. 2025;15(1):4180.
91. Eldegwey ZG, EL-Nahas NG, Draz RS, Gendia WM. Effect of Virtual Reality on Balance in Elderly with Type 2 Diabetes Mellitus. The Egyptian Journal of Hospital Medicine (April 2025).99:1650-5.

92. Elgayar SL, Tolba AM, Elhamrawy MY. Effect of High Intensity Focused Electromagnetic Therapy on Balance and Quality of Life in Elderly Type 2 Diabetic Men: A Randomized Controlled Trial. Archives of physical medicine and rehabilitation. 2025.
93. Elsaid NB, Barakat AM, Abdelwanees S. Fear Of Falling and Associated Factors among Elderly People Attending Outpatients Clinics at Menoufia University Hospital. The Egyptian Family Medicine Journal. 2024;8(1):3-20.
94. Elshoura HMI, Alashry AMA, Moussa SG. Role of balance exercise dynamic stress in rehabilitation of heart failure patients with reduced ejection fraction. EGYPTIAN RHEUMATOLOGY AND REHABILITATION. 2025;52(1).
95. Fatima SN, Tanveer F, Shoukat F, Ahmad A, Siddique K. Effects of balance training with and without gaze stabilization exercises on clinical outcomes in elderly patients with chronic dizziness: A randomized controlled trial. Journal of Bodywork and Movement Therapies. 2022;32:46-50.
96. Ferhi H, Maktouf W. The impact of obesity on static and proactive balance and gait patterns in sarcopenic older adults: an analytical cross-sectional investigation. Peerj. 2023;11.
97. Ghous M, Masood Q, Malik AN, Afridi A, Mehmood Q. Comparison of Nonimmersive Virtual Reality and Task-Oriented Circuit Training on Gait, Balance and Cognition Among Elderly Population: A Single-Blind Randomized Control Trial. Games for Health Journal. 2024;13(3):164-71.
98. Hassan Ahmed Saleh S, Wessam Aly W, Gamal Saber H, Kamel Abdel Aziz B. Falls amongst elderly attending the outpatient's clinic in the Geriatric medicine, in Ain Shams University Hospitals: Prevalence and risk factors. The Egyptian Journal of Geriatrics and Gerontology. 2024;11(1):12-24.
99. Hussein MM, Hamza SA, Fouad DA, Aly WW, Marawan DA, Elsorady KE. Association Between Serum Osteocalcin Levels with Frailty, Risk of Falls and Glycemic Control Among Elderly Diabetic Patients. Aging Medicine and Healthcare. 2024;15(2):47-53.
100. Kandeel SEA, Taha HM, Sweed HS, Abdelrahman TT. Risk of falls in the elderly and its relation with vestibular and cognitive function. Ain Shams Medical Journal. 2024;75(2):425-32.
101. Kaynat F, Nazir S, Kumail SM, Ramal S, Arooj A, Farooq A. Association between Balance Confidence and Risk of Fall among Geriatric Population. Indus Journal of Bioscience Research. 2025;3(1):533-8.
102. Khalil MIM, Ashour A, Shaala RS, Mousa EFS, Sorour DM. Effect of a digital two-dimensional animation program on attitude toward walking aids, performance, and fall avoidance behaviors among older adults in assisted living facilities. Geriatric Nursing. 2024;60:32-41.
103. Khallaf M, Jaber H, Alameri M, Magdy D, Kamal H, Hassanin M, et al. Effect of Vestibular-Oriented Balance Training on Postural Control and Risk of Fall in Patients With Parkinson's Disease. Neurology Research International. 2025;2025(1):6846267.
104. Magtouf E, Chortane SG, Chortane OG, Boyas S, Beaune B, Durand S, et al. Influence of Concurrent Exercise Training on Ankle Muscle Activation during Static and Proactive Postural Control on Older Adults with Sarcopenic Obesity: A Multicenter, Randomized, and Controlled Trial. European Journal of Investigation in Health Psychology and Education. 2023;13(12):2779-94.
105. Mousa SM, Rasheedy D, El-Sorady KE, Mortagy AK. Beyond mobility assessment: Timed up and go test and its relationship to osteoporosis and fracture risk. Journal of Clinical Gerontology & Geriatrics.7(2):48-52.
106. Nazeer R, Asai T, Tantisuwat A. Association of balance, anthropometric measurements, fall risk, and awareness with quality of life in older females according to hypertensive status. Medicine (Baltimore). 2025;104(35):e44210.
107. Sayed EAG, Abou-Raya A, El-Bordiny M, Yehia K. Clinical, Laboratory Characteristics, Comorbidity and Function in Elderly patients with Rheumatoid Arthritis. SVU-International Journal of Medical Sciences. 2025;8(1):27-38.
108. Waheed A, Kashif M, Ghaffar T, Khalid M, Afzal S, Asif R. Reliability and Validity of Urdu Version of the Dynamic Gait Index in Pakistani Geriatric Population. Journal of the Liaquat University of Medical and Health Sciences. 2024;23(1):63-8.

109. Zahra S, Waris M, ul Ain Q, Sajjad Y. Normative data of modified Romberg balance test for risk of fall in elderly population of Pakistan. Journal of the Pakistan Medical Association. 2023;73(3):515-9.
110. Hoy D, Brooks P, Woolf A, Blyth F, March L, Bain C, et al. Assessing risk of bias in prevalence studies: modification of an existing tool and evidence of interrater agreement. J Clin Epidemiol. 2012;65(9):934-9.
111. Furuya-Kanamori L, Barendregt JJ, Doi SAR. A new improved graphical and quantitative method for detecting bias in meta-analysis. JBI Evidence Implementation. 2018;16(4):195-203.
